# Morphological Stability for in silico Models of Avascular Tumors

**DOI:** 10.1007/s11538-024-01297-x

**Published:** 2024-05-17

**Authors:** Erik Blom, Stefan Engblom

**Affiliations:** Division of Scientific Computing, Department of Information Technology, Uppsala University, 751 05, Uppsala, Sweden

**Keywords:** Computational tumorigenesis, Cell population modeling, Emergent property, Darcy’s law, Saffman–Taylor instability, Primary 35B35, 92C10, Secondary 65C40, 92-08

## Abstract

The landscape of computational modeling in cancer systems biology is diverse, offering a spectrum of models and frameworks, each with its own trade-offs and advantages. Ideally, models are meant to be useful in refining hypotheses, to sharpen experimental procedures and, in the longer run, even for applications in personalized medicine. One of the greatest challenges is to balance model realism and detail with experimental data to eventually produce useful data-driven models. We contribute to this quest by developing a transparent, highly parsimonious, first principle in silico model of a growing avascular tumor. We initially formulate the physiological considerations and the specific model within a stochastic cell-based framework. We next formulate a corresponding mean-field model using partial differential equations which is amenable to mathematical analysis. Despite a few notable differences between the two models, we are in this way able to successfully detail the impact of all parameters in the stability of the growth process and on the eventual tumor fate of the stochastic model. This facilitates the deduction of Bayesian priors for a given situation, but also provides important insights into the underlying mechanism of tumor growth and progression. Although the resulting model framework is relatively simple and transparent, it can still reproduce the full range of known emergent behavior. We identify a novel model instability arising from nutrient starvation and we also discuss additional insight concerning possible model additions and the effects of those. Thanks to the framework’s flexibility, such additions can be readily included whenever the relevant data become available.

## Introduction

Tumors are highly complicated biological systems, yet constitute a concrete example of cellular self-organization processes amenable to modeling in silico (Brú et al. 2003). In a cancerous tumor, the cells have undergone several significant mutations and obtained distinct *hallmarks* providing the population with remarkable growth capabilities Hanahan and Weinberg (2000, 2011). Furthermore, populations comprising large numbers of cells interact on multiple scales, yielding a range of emergent phenomena (Deisboeck and Couzin 2009), which can be studied using computational models based on knowledge of single-cell behavior. To the modeler’s aid in this regard, biological data streams nowadays contain detailed features at the individual cell level such as cell size and -type, mutation- and growth rate, molecular constituents, and gene expression (Saadatpour et al. 2015; Cermak et al. 2016; Anderson and Dittrich 2006).

Complementing biological experiments, mathematical models can in addition provide explanations to observed data, concerning, e.g., drug-response in tumor growth, with potential applications in precision medicine (Yin et al. 2019; Barbolosi et al. 2016). Progress in cell biology has led to a good understanding of intra-cellular processes which unlocks the possibility to model these systems from fundamental principles, to translate ‘word models’ formulated from biological experiments into mathematical and computational models, and to test the features of these models (Roose et al. 2007). Often quoted uses of computational models include the testing of hypotheses, the investigation of causality, and the integration of knowledge when comparing in vitro and in vivo data (Brodland 2015). Bayesian inference methods present a means to quantitatively investigate these matters, provided there exist appropriate data and meaningful priors associated with the model parameters.

Several cell population models exist in the literature, ranging from continuous to agent-based to hybrid models, and taking place at various scales (Schlüter et al. 2015; Fletcher et al. 2013; Szabó and Merks 2013; Deisboeck 2011; Cristini et al. 2003). Such models may reach a predictive power, where agreement/disagreement with biological data can advance our understanding of mechanistic relations within the biological systems (Jin 2016; Frieboes et al. 2009; Bearer 2009; Drasdo and Höhme 2005). Pertinent to the present work, previous research shows how analyzing the emergent morphology of cell population models can provide insight into the role of the model parameters (Giverso et al. 2016; Gerlee and Anderson 2007; Anderson et al. 2006), promoting future use of Bayesian methods. Such analysis has, for example, enabled modelers to analyze the behavior of the invasive fronts of tumor models and their response to parameter changes representing vascularization, nutrient availability (Cristini et al. 2005; Lowengrub et al. 2009), and cell-cell adhesion effects (Byrne and Chaplain 1996; Scianna and Preziosi 2012).

Motivated in part by improvements of in vitro techniques for obtaining detailed time-series tumor and single-cell data and the current trend in computational science towards data-driven modeling, we present and analyze a basic continuous mathematical model of avascular tumor growth, here derived from a previously developed cell-based model (Engblom et al. 2018). Our aim is that the model should be highly parsimonious in order to cope with issues of model identifiability. For this purpose the in silico tumor’s fate should be well understood when regarded as a map from parameters to simulation end-result. Initial results from an earlier version of the model (Engblom et al. 2018) display boundary instabilities, akin to those discussed in Greenspan (1976), which we analyze thoroughly. The self-regulating properties of avascular tumors concerning size that have been observed in vitro (Folkman and Hochberg 1973) and in silico (Grimes et al. 2016) motivate a careful investigation into the model capabilities in this regard.

We have structured the paper as follows. In Sect. 2 we summarize the stochastic cell-based tumor model as well as the associated mean-field space-continuous version. We analyze the latter in Sect. 3, assuming radially symmetric solutions first, and then via linear stability analysis. In Sect. 4 we investigate via numerical examples the key aspects of the analysis as well as its relevance for the stochastic model. A concluding discussion is found in Sect. 5.

## Stochastic Modeling of Avascular Tumors

An advantage held by stochastic models is that they implicitly define a consistent likelihood and thus formally have the potential to be employed in Bayesian modeling when confronted with data. We summarize our stochastic framework in Sect. 2.1 and the basic stochastic tumor model in Sect. 2.2. We next derive a corresponding mean-field version in Sect. 2.3, which has the distinct advantage of being open to mathematical analyses.

### Stochastic Framework

The Darcy Law Cell Mechanics (DLCM) framework (Engblom et al. 2018) is a cell-based stochastic modeling framework where the cells are explicitly represented and the rates of their state updates, e.g., movement, proliferation, death, etc., are determined and govern the corresponding events in a continuous-time Markov chain. Movements of the cells generally follow *Darcy’s law* for fluid flow in a porous environment, but since the framework takes place in continuous time, other types of cell transport are easily incorporated.

**Fig. 1:**
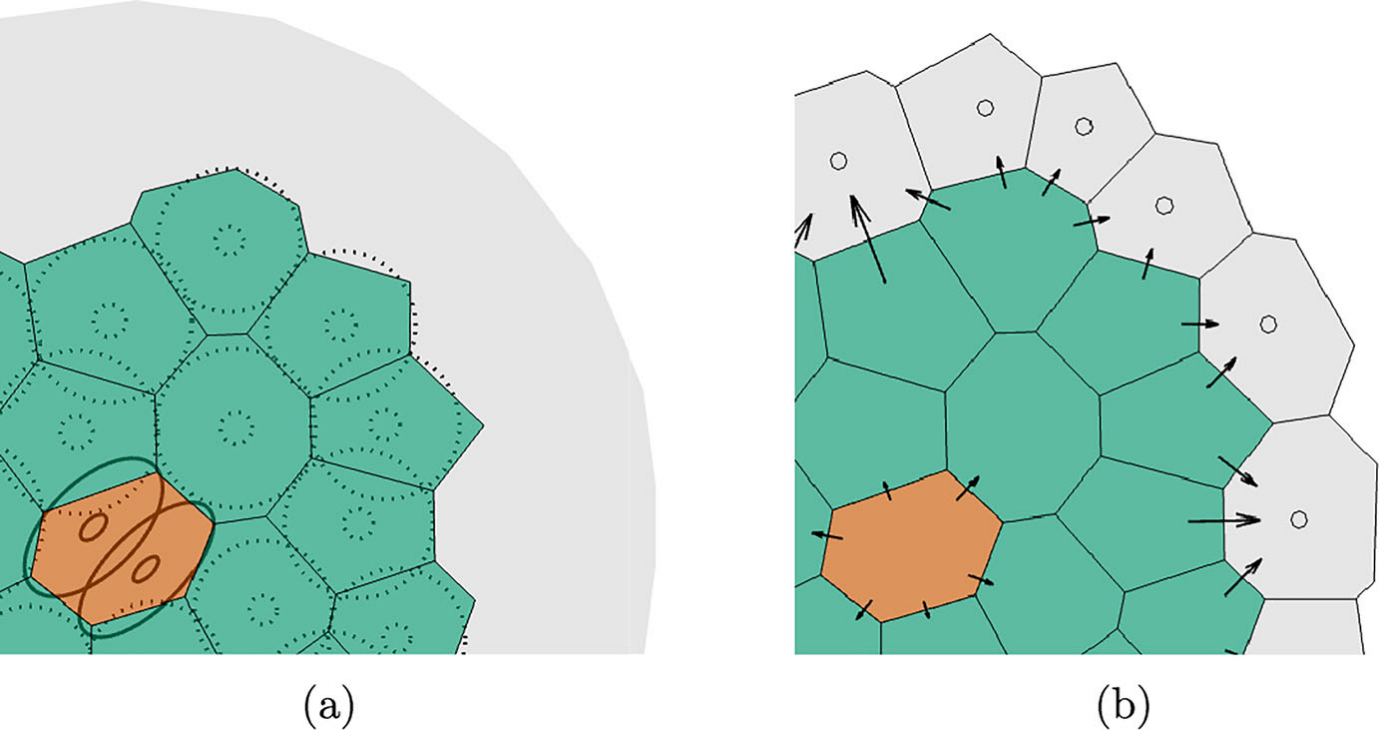
Cell population representation in the DLCM framework using a Voronoi tessellation. Green voxels represent singly occupied voxels and red voxels represent doubly occupied voxels as shown explicitly in (**a**), where the ellipses indicate the corresponding underlying population of cells. The grey area highlights a region of empty voxels. The doubly occupied voxels exceed the carrying capacity and exert pressure on the surrounding cells. The movement rates are visualized in (**b**), where the arrows represent movement rate and direction for a subset of the possible movements. The grey voxels represent empty voxels that cells may migrate into. Adapted from Engblom et al. (2018), Fig. 2.1

The spatial domain is discretized into $i = 1,2,..., N_{\textrm{vox}}$ voxels $v_i$, and populated by a total of $N_{\textrm{cells}}$ cells. The DLCM framework can be used over any grid for which a consistent discrete Laplace operator can be derived. Each voxel may be empty or contain some number of cells and if this number exceeds the voxel’s *carrying capacity*, the cells will exert a pressure onto the cells in the surrounding voxels, see Fig. 1. The pressure propagates through the considered domain and the local pressure gradient induces a cell flow. The simplest implementation allows each voxel to be populated by $u_i \in \{0, 1, 2\}$ cells at any time, thus with a carrying capacity of 1. Note that carrying capacity here does not refer to the maximum possible number of cells in a voxel, but rather to the capacity beyond which a voxel is no longer in a mechanically relaxed state.

Although the state $u_i$ takes on discrete values in each voxel and at any given time, the governing model is derived from a continuous assumptions where the corresponding cell density is then $u = u(x,t)$. We let *u* be governed by the continuity equation

2.1 $$\begin{aligned} \frac{\partial u}{\partial t} + \nabla \cdot I = 0, \end{aligned}$$

where *I* is the flux of *u*. There are three main assumptions in the DLCM framework, with the first assumption pointing to the central role of the flux *I*:

#### Assumption 2.1

Consider the discrete tissue formed from the population of cells distributed over the grid. We assume that:

1. The tissue is in mechanical equilibrium when all cells are placed in a voxel of their own.
2. The cellular pressure of the tissue relaxes rapidly to equilibrium in comparison with any other mechanical processes of the system.
3. The cells in a voxel occupied by *n* cells may only move into a neighboring voxel if it is occupied by less than *n* cells.

From Assumption 2.1(1)it is clear that random (e.g., Brownian) motion around a voxel center is ignored, and, therefore, that only voxels with cells above the carrying capacity are considered pressure sources. As in Engblom et al. (2018), the flux is determined from the pressure gradient in the form of Darcy’s law which can be derived as a limit for flow through porous media (Whitaker 1986):

2.2 $$\begin{aligned} I = -D \nabla p, \end{aligned}$$

where *p* is the pressure and the Darcy constant *D* can be interpreted as the ratio of the medium permeability $\kappa$ to its dynamic viscosity $\mu$, $D:= \kappa / \mu$.

The relation between pressure and cell population is completed by assuming a constitutive relation in the form of a heat equation for the pressure and using Assumption 2.1(2) to arrive at the stationary relation

2.3 $$\begin{aligned} -\Delta p = s(u), \end{aligned}$$

where *s*(*u*) is the pressure source which equals to one for voxels above the carrying capacity, $u_i > 1$, and zero otherwise.

Finally, we detail the flux parameter *D* in (2.2). Let *R*(*e*) denote the rate of an event *e*, and let $i \rightarrow j$ denote the event that a cell moves from voxel $v_i$ to $v_j$. With unit carrying capacity only two distinct movements rates are possible according to Assumption 2.1(3): one for cells moving into an empty voxel, and one for cells moving into an already occupied voxel,

2.4 $$\begin{aligned} \left. \begin{array}{rcl} R(i \rightarrow j; \quad u_i \ge 1, \quad u_j = 0) & =& D_1 I(i \rightarrow j)\\ R(i \rightarrow j; \quad u_i > 1, \quad u_j = 1) & =& D_2 I(i \rightarrow j) \end{array} \right\} \end{aligned}$$

where $D_1$ and $D_2$ are (possibly equal) conversion factors from units of pressure gradient to movement rate for the respective case. Here, $I(i \rightarrow j)$ is the pressure gradient integrated over the boundary shared between the voxels $v_i$ and $v_j$. To enable a comparison of the DLCM model with a corresponding PDE model the effect of surface tension needs to be included. As a consequence of this, we also need to include migration between neighboring singly occupied voxels on boundaries where surface tension is added, and then with a rate coefficient equal to $D_1$. This is an event which is formally not allowed in the original framework, cf. Engblom et al. (2018).

To sum up, a population of cells occupying a grid may be evolved in time by first solving for the pressure in (2.3) using the Laplacian on the grid, and then converting the pressure gradient into rates via (2.2) and (2.4). The rates are now interpreted as competing Poissonian events for the corresponding cellular movements to be simulated as a continuous-time Markov chain. Any other dynamics taking place in continuous time are thus readily incorporated in a consistent way.

### Framework Tumor Model

As a candidate for a ‘minimal’ avascular tumor model we consider the one presented in Engblom et al. (2018) which consists of a single cancerous cell type in three different states: *proliferating*, *quiescent* (i.e., dormant), and *necrotic* cells. As a matter of convenient implementation the range of $u_i$ can be extended to include $u_i = -1$ which represents a voxel containing a dead necrotic cell so that $u_i \in \{ -1, 0, 1, 2\}$. Also, let $\Omega$ denote the tumor domain with $u_i \ne 0$ and let $\Omega _{\textrm{ext}}$ denote the entire computational domain.

An avascular tumor has to rely on oxygen and nutrients to diffuse through the surrounding tissue to reach the tumor, a process assumed to be much faster than cell migration, growth, and death. As such, nutrients are readily modeled by a stationary heat equation with a boundary condition on the external boundary $\partial \Omega _{\textrm{ext}}$ (far away from the tumor boundary $\partial \Omega$) as

2.5 $$\begin{aligned} \left. \begin{array}{rcl} -\Delta c & =& - \lambda a(u) \\ c & =& c_{\textrm{out}}\quad \text { on } \partial \Omega _{\textrm{ext}}\end{array} \right\} \end{aligned}$$

where *c* is the concentration variable understood as a proxy variable for oxygen and any other nutrients required for the cellular metabolism. Further, $\lambda$ is the ratio of the oxygen consumption rate to the oxygen diffusion rate, and $a(u_i)$ is the number of living cells in the voxel *i*, i.e., $a(u_i) = \max (u_i,0)$. The rates describing the tumor growth are then defined as follows: cells are in the proliferating state if $c_i \ge \kappa _{\textrm{prol}}$ and then divide at rate $\mu _{\textrm{prol}}$, where $\kappa _{\textrm{prol}}$ is the minimum oxygen concentration required for cell proliferation. A cell dies and then becomes necrotic at rate $\mu _{\textrm{death}}$ if $c_i < \kappa _{\textrm{death}}$, where $\kappa _{\textrm{death}}$ is the minimum oxygen concentration required for individual cell survival. Finally, necrotic cells degrade at rate $\mu _{\textrm{deg}}$ to free up the voxel they are in. Cells in voxels at intermediate oxygen levels are in the quiescent state. Note that cells instantly switch between all living states provided the oxygen concentration allows for it.

At the tumor boundary a pressure condition needs to be imposed in order to capture the net effect of cell-cell adhesion as well as the interactions between cancerous and healthy cells. We let the phenomenological constant $\sigma$ represent this via a Young-Laplace pressure drop proportional to the boundary curvature *C*. Denoting by $p^{(\textrm{ext})}$ the ambient pressure outside the tumor (i.e., in $\Omega _{\textrm{ext}}\setminus \Omega$) we thus have the Dirichlet condition

2.6 $$\begin{aligned} p = p^{(\textrm{ext})}- \sigma C, \qquad \text { on } \partial \Omega . \end{aligned}$$

An alternative approach to (2.6) for including surface tension effects directly on the microscopic level can be found in Engblom et al. (2018), where the local adhesive forces work passively to resist cell migration directly by negative contributions to (2.4). Local implementations of adhesion that do not change the population pressure field as with (2.6) are unfortunately not fully consistent with a pressure-driven migration law, thus obscuring a mechanistic understanding. The implementation of (2.6) along with other modifications to the DLCM framework are further discussed in Sect. B.

A summary of the parameters of the proposed model is found in Table 1.

**Table 1:** Parameters of the cell-based tumor model in two dimensions. The same parameters are used in the corresponding PDE model (except for $\mu _{\textrm{deg}}$ which is not used) and are nonnegative for both models. The units *t*, *l*, *f* correspond to units of time, length, and force, respectively

Parameter	Description
*D*	Ratio medium permeability to dynamic viscosity $[f^{-1}l^{3}t^{-1}]$
$\lambda$	Ratio of oxygen consumption to diffusion rate $[l^{-2}]$
$c_{\textrm{out}}$	Oxygen concentration at oxygen source $[l^{-2}]$
$\mu _{\textrm{prol}}$	Rate of cell proliferation $[t^{-1}]$
$\mu _{\textrm{death}}$	Rate of cell death $[t^{-1}]$
$\mu _{\textrm{deg}}$	Rate of dead cell degradation $[t^{-1}]$
$\kappa _{\textrm{prol}}$	Oxygen concentration threshold for cell proliferation $[l^{-2}]$
$\kappa _{\textrm{death}}$	Oxygen concentration threshold for cell death $[l^{-2}]$
$\sigma$	Surface tension coefficient [*f*]

### Mean-field PDE Tumor Model

We next derive the corresponding ‘minimal’ partial differential equations (PDE) model, constructed to very closely mimic the mean-field of the stochastic model. Certain aspects of the DLCM model, e.g., the exclusion principle in (2.4), which excludes migration events between neighboring singly occupied voxels, but also (2.5), where a nonlinear interaction between nutrients and the cell population takes place, invalidate the assumption of independence between the states of different voxels, cf. Davies (2014). This complicates formulating a mean-field PDE exactly. However, the underlying continuous physics of the DLCM model provides an appropriate starting point for the construction of a corresponding PDE model. The derivation is essentially based on mass balance with a cellular growth- and death rates, and a velocity field proportional to the pressure gradient.

Seeing as cells comprise mostly water we assume that they are incompressible. This implies that the material derivative is zero, and hence the conservation law governing the tumor cell density $\rho$ in a velocity field $\varvec{v}$ becomes

2.7 $$\begin{aligned} \frac{\partial \rho }{\partial t} + \nabla \cdot (\varvec{v} \rho ) = \underbrace{\frac{\partial \rho }{\partial t} + \varvec{v} \cdot \nabla \rho }_{= 0 \text { by incompressibility}} + \rho \nabla \cdot \varvec{v} = \Gamma , \end{aligned}$$

where $\Gamma$ is cell growth and loss due to proliferation or death, respectively, and remains to be defined. Assuming that the cells move as a viscous fluid with low Reynolds number through a porous medium, the velocity field for the cell density is governed by Darcy’s law (Whitaker 1986)

2.8 $$\begin{aligned} \varvec{v} = - D \nabla p, \end{aligned}$$

where the porous media that the cells reside in is the extra-cellular matrix (ECM), whose permeability is one of two factors determining *D*. Combining (2.7) and (2.8) and assuming a homogeneous permeability within the tissue domain we arrive at

2.9 $$\begin{aligned} - \rho \Delta p = \frac{\Gamma }{D}. \end{aligned}$$

For the source term $\Gamma$ we mimic the stochastic model and let the cellular growth and death rates be constant and determined by oxygen thresholds. We thus define $\Gamma$ as

**Fig. 2:**
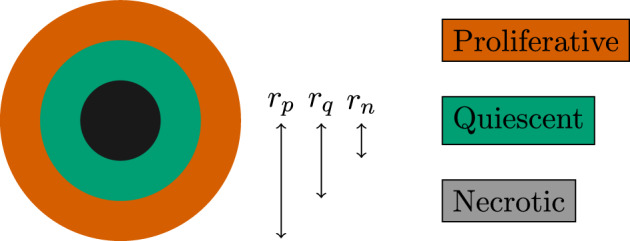
Schematic of circular tumor with three distinct regions. Arrows show the distinct radii, $r_p$, $r_q$, and $r_n$ which correspond to the distance from the origin to respective regional interface

2.10 $$\begin{aligned} \Gamma = {\left\{ \begin{array}{ll} \mu _{\textrm{prol}}\times \rho & c \ge \kappa _{\textrm{prol}}\\ 0 & \kappa _{\textrm{death}}\le c< \kappa _{\textrm{prol}}\\ -\mu _{\textrm{death}}\times \rho & c < \kappa _{\textrm{death}}, \end{array}\right. } \end{aligned}$$

which defines the proliferative, quiescent, and necrotic region, respectively (see Fig. 2). These relations close the pressure relation (2.9).

Similar to the cell-based formulation, we assume a Young-Laplace pressure drop at the outer tumor boundary, which obeys (2.6). This surface tension effect arises from various cell-cell adhesion effects—the loss of which, due to loss of E-cadherin function in cells, is associated with tumor metastasis and tissue invasion (Hanahan and Weinberg 2000). Let $\Omega$ here denote the region with $\rho > 0$ akin to the tumor domain of the stochastic model. By Darcy’s law, we then have that $\partial \Omega ^-$ moves with the velocity

2.11 $$\begin{aligned} \varvec{v}_{\partial \Omega ^-} = (-D\nabla p) \cdot \varvec{n}, \end{aligned}$$

where $\varvec{n}$ is the interface normal vector and $\partial \Omega ^-(t)$ denotes the boundary as approached from inside $\Omega$. The condition (2.11) connects the velocity field with the movement of the tumor boundary.

The pressure boundary conditions concern the external medium that the tumor grows within. Recall that $\Omega _{\textrm{ext}}$ is the domain containing both $\Omega$ and the external medium. By assuming that the external medium obeys laws similar to the tumor tissue, we can summarize the medium’s impact on the tumor growth through the tumor boundary conditions. We start from the assumption that the pressure propagates freely throughout the external medium outside the tumor and, for consistency of the complete two-tissue system, the external medium is assumed to abide by the same assumptions as the tumor tissue (i.e., incompressibility, Darcy’s law, and conservation of mass). We further assume that growth and death of the external tissue is negligible and, thus, arrive at the governing equation for the outer pressure, $p^{(\textrm{ext})}$ in $\Omega _{\textrm{ext}}\setminus \Omega$:

2.12 $$\begin{aligned} \Delta p^{(\textrm{ext})}(\varvec{r})= &  0, \end{aligned}$$

where the solution is undetermined up to a specified boundary condition. The velocities of the tumor and the external tissue are both determined by Darcy’s law, but are allowed different Darcy coefficients, *D* and $D_{\textrm{ext}}$, respectively. The velocities are assumed compatible at the interface and the complete set of boundary conditions on $\partial \Omega$ thus reads as

2.13 $$\begin{aligned} p&= p^{(\textrm{ext})}- \sigma C, \end{aligned}$$

2.14 $$\begin{aligned} \varvec{n} \cdot D\nabla p&= \varvec{n} \cdot D_{\textrm{ext}}\nabla p^{(\textrm{ext})}. \end{aligned}$$

Thus, we assume that the physical extent of the region between the tissues (where cell mixing might occur) is negligible in comparison with the spatial scale of the model. The nondimensional coefficient is expressed as $\hat{D}_{\text {ext}} = D_{\textrm{ext}}/D$, but for brevity we omit the hat in the following analysis. The compatibility condition (2.14) allows for an investigation into the effects of varying the stiffness between the tumor and the external tissue due to, e.g., an increase in collagen density (Valero et al. 2018). For simplicity, we assume that the ECM permeability is homogeneous and time-independent across the domain of tissues, hence implicitly assuming that breakdown and remodeling of ECM that could affect tumor progression (Quail and Joyce 2013) are negligible.

Finally, oxygen diffuses in towards the tumor through the surrounding tissue from a source (e.g., a vessel) far away with regards to the spatial scale of the system. Akin to the cell-based model, we consider a stationary heat equation but with a different source term as

2.15 $$\begin{aligned} - \Delta c = {\left\{ \begin{array}{ll} -\lambda \rho , \quad & c \ge \kappa _{\textrm{death}}\\ 0, \quad & c < \kappa _{\textrm{death}}\text { on } \Omega _{\textrm{ext}}\\ \end{array}\right. } \end{aligned}$$

with $c = c_{\textrm{out}}$ on $\partial \Omega _{\textrm{ext}}$, and where $\lambda$ is ratio of the consumption rate per cell *density* to oxygen diffusion rate in the PDE setting. We assume that $\partial \Omega _{\textrm{ext}}$ is radially symmetric and lies at a distance *R* from the domain origin and we also make the simplifying assumption that the external tissue consumes negligible oxygen.

A suitable choice for the characteristic length is $l_c = R$ as it remains constant during growth. Nondimensionalization of the model assuming a radially symmetric tumor then yields the characteristic units

2.16 $$\begin{aligned} l_c = R, \quad v_c = \mu _{\textrm{prol}}R, \quad t_c = 1/\mu _{\textrm{prol}}, \quad p_c = \mu _{\textrm{prol}}R^2/D, \end{aligned}$$

with the dimensionless parameters $\hat{\sigma } = D/(\mu _{\textrm{prol}}R^3) \times \sigma$ and $\hat{\mu }_{\text {death}} = \mu _{\textrm{death}}/ \mu _{\textrm{prol}}$. We nondimensionalize the oxygen parameters independently of the pressure and arrive at the characteristic units and additional dimensionless parameter, respectively, as

2.17 $$\begin{aligned} \left. \begin{array}{l} \quad c_c = c_{\textrm{out}}, \quad \hat{\lambda } = {\lambda /c_{\textrm{out}}}, \\ \hat{\kappa }_{\text {prol}} = \kappa _{\textrm{prol}}/ c_{\textrm{out}}, \quad \hat{\kappa }_{\text {death}} = \kappa _{\textrm{death}}/ c_{\textrm{out}}. \end{array} \right\} \end{aligned}$$

Subsequently, we use the characteristic units and nondimensional parameters, but we drop the hats. The units are set to one such that $R = 1$, $\mu _{\textrm{prol}}= 1$, $c_{\textrm{out}}= 1$, and $D = 1$.

While the PDE model is intended to closely match the mean-field of the DLCM model, there exist certain differences between the two and we view the PDE model as an *effective* model of the DLCM model. For the simulation of the PDE model, we thus use effective parameters that we derive from the outcome of the DLCM simulations (details in Sect. 4).

## Analysis

We analyze the morphological properties of the tumor model in two spatial dimensions. This simplifies the analysis while still allowing for a qualitative comparison with in vitro data from tumor spheroids embedded in matrigel, where nutrients enter through the spherical surface (corresponding to the circular boundary in 2D). The case of a radially symmetric growth is discussed in Sect. 3.1 and a spatial linear stability analysis in Sect. 3.2. In essence, the outcome of the latter analysis include conditions for when the former radially symmetric case is a valid ansatz. Finally, in Sect. 3.3 we uncover how the morphological instabilities of the model develop during its different growth phases and a possible role of the external medium in exacerbating or reducing such effects.

### Radial Symmetry and the Stationary State

A characteristic property of the avascular tumor is that it reaches a stationary growth phase due to limited oxygen/nutrition availability (Folkman and Hochberg 1973). We therefore first derive analytical relations that provide insight into which regions of the model parameter space map to such a stationary state under the preliminary assumption that the tumor is radially symmetric.

The constant cellular growth and death rates define distinct characteristic regions of the model tumor according to (2.10): the proliferative, quiescent, and necrotic region. For a radially symmetric tumor at time $t \ge 0$, motivated by the form of the oxygen field governed by (2.15), we let $r_p(t)$ denote the tumor radius, $r_q(t)$ the radius of the interface between the proliferative and quiescent region, and $r_n(t)$ the radius of the interface between the quiescent and necrotic region, cf. Fig. 2. Given $\lambda > 0$, the assumed radial symmetry implies that $0 \le r_n \le r_q \le r_p$, and under the chosen units, $r_p < R = 1$. We note that if $r_p$ is sufficiently close to the oxygen source at *R*, the model assumption of avascularity breaks down.

We simplify the problem by assuming that $\rho = 1$ across the entire tumor domain since this allows the oxygen field to be explicitly solved. By incompressibility and slow migration of cells, this is a reasonable approximation and, besides, a constant cell density in the PDE model is a close match to the discrete stochastic model that we wish to investigate.

We thus solve (2.15) under radial symmetry while imposing $C^1$-continuity for the oxygen *c* across the interfaces between the characteristic regions. Under radial symmetry, the divergence theorem applied to (2.15) implies an inhomogeneous Neumann boundary condition across each radial boundary whose value is proportional to the volume of oxygen sinks contained within. The full problem for the oxygen under radial symmetry thus reads

3.1 $$\begin{aligned} \begin{aligned} -\frac{\partial }{r \partial r} \bigg ( \frac{r \partial c }{ \partial r}\bigg ) = s_c(r) := {\left\{ \begin{array}{ll} -\lambda , \quad & r_{n} \le r \le r_{p}, \\ 0, \quad & \text {otherwise} \end{array}\right. } \\ c(1) = 1, \quad c'(r_i) = -\frac{1}{r_i}\int _0^{r_i} s_c(s)s \, ds, \; \text{ for } r_i \in \{r_{n}, r_{q}, r_{p}\}, \end{aligned} \end{aligned}$$

where the last three relations follow from application of the divergence theorem under radial symmetry and at each interface separately. We solve (3.1) and find

3.2 $$\begin{aligned} c(r)&= \left\{ \begin{array}{ll} 1 + \lambda /2 \, \left( r_p^2- r_n^2 \right) \log r, & r_p \le r,\\ 1 + \lambda /2 \, \left( r_p^2 \log r_p -r_n^2 \log r + (r^2 - r_p^2)/2 \right) , & r_n \le r \le r_p, \\ \kappa _{\textrm{death}}, & r \le r_n, \end{array} \right. \end{aligned}$$

where the expression does not differ in the proliferative and quiescent regions since the oxygen consumption rates are identical there. By definition, the oxygen level at $r_q$ is $\kappa _{\textrm{prol}}$ and at $r_n$ it is $\kappa _{\textrm{death}}$. Using this, (3.2) implies the following algebraic relations between the characteristic regions

3.3 $$\begin{aligned} \left. \begin{array}{rclcl} K_{\textrm{prol}}& :=& 4(1-\kappa _{\textrm{prol}})/\lambda & =& -r_p^{2} \log r_p^2+ r_n^2 \log r_q^2 - r_q^2 + r_p^{2} \\ K_{\textrm{death}}& :=& 4(1-\kappa _{\textrm{death}})/\lambda & =& -r_p^{2} \log r_p^2 + r_n^2 \log r_n^2 -r_n^2 + r_p^{2} \end{array} \right\} \end{aligned}$$

in terms of the reduced parameter set $\{K_{\textrm{prol}},K_{\textrm{death}}\}$.

Given radial symmetry and $\rho = 1$, the tumor volume change is derived from mass balance as $\dot{V} = \mu _{\textrm{prol}}V_p - \mu _{\textrm{death}}V_n$, where $V_p$ and $V_n$ are the volumes of the proliferative and necrotic region, respectively. Thus, in two spatial dimensions we get that

3.4 $$\begin{aligned} d/dt \, \left( r_p^2 \right) = -\mu _{\textrm{death}}r_n^2 - r_q^2 + r_p^{2}, \end{aligned}$$

under the nondimensionalization where $\mu _{\textrm{prol}}= 1$. Hence an initial state $r_p(t = 0)$ together with the reduced parameter set $\{K_{\textrm{prol}},K_{\textrm{death}},\mu _{\textrm{death}}\}$ fully determine the dynamics of a radially symmetric tumor under (3.3)–(3.4).

Assume now that $(r_n^{\textrm{eq}},r_q^{\textrm{eq}},r_p^{\textrm{eq}})$ is a stationary solution of (3.3)–(3.4). Writing $r_{q}^2 = r_{q}^2(r_{p}^2)$ and $r_{n}^2 = r_{n}^2(r_{p}^2)$, and by implicitly differentiating (3.3) we can linearize (3.4) around this solution and retrieve the single eigenvalue

3.5 $$\begin{aligned} \Lambda _r&= 1 - \frac{\log r_p^{\textrm{eq}}}{\log r_n^{\textrm{eq}}} \left( \mu _{\textrm{death}}+ \frac{2 \log \frac{r_q^{\textrm{eq}}}{r_n^{\textrm{eq}}}}{(r_q^{\textrm{eq}})^2 - (r_n^{\textrm{eq}})^2} \, (r_q^{\textrm{eq}})^2\right) . \end{aligned}$$

#### Proposition 3.1

(*Stability of radially symmetric equilibrium*) Given $\mu _{\textrm{death}}> 0$, assume that $0 \le r_n^{\textrm{eq}}\le r_q^{\textrm{eq}}\le r_p^{\textrm{eq}}$ is a stationary solution of (3.3)–(3.4). Then $\Lambda _r < 0$ in (3.5) whenever $r_p^{\textrm{eq}}\le \exp (-1) \approx 0.368$.

#### Proof

Put $(r_n^{\textrm{eq}})^2 = \eta (r_q^{\textrm{eq}})^2$ for some $\eta \in (0,1)$ and note that $(r_p^{\textrm{eq}})^2 = (1+\mu _{\textrm{death}}\eta ) (r_q^{\textrm{eq}})^2$ by stationarity (the cases $\eta \in \{0,1\}$ are treated as limits). The eigenvalue becomes

$$\begin{aligned} \Lambda _r&= 1 - \frac{\log r_p^{\textrm{eq}}}{\log r_p^{\textrm{eq}}+\frac{1}{2} \log \left( \eta /(1+\mu _{\textrm{death}}\eta ) \right) } \left( \mu _{\textrm{death}}- \frac{\log \eta }{1-\eta } \right) . \end{aligned}$$

By inspection we find that as a function of $\mu _{\textrm{death}}$, the expression on the right is monotonically decreasing and hence it is bounded by its behavior as $\mu _{\textrm{death}}\rightarrow 0+$:

$$\begin{aligned} \Lambda _r&< 1 + \frac{\log r_p^{\textrm{eq}}}{\log r_p^{\textrm{eq}}+\frac{1}{2} \log \eta } \times \frac{\log \eta }{1-\eta }. \end{aligned}$$

In turn, as a function of $r_p^{\textrm{eq}}$, this expression is monotonically increasing such that, in particular, for $r_p^{\textrm{eq}}\le \exp (-1)$ we have that

$$\begin{aligned} \Lambda _r&< 1 + \frac{\log \eta }{1-\frac{1}{2} \log \eta } \times \frac{1}{1-\eta }. \end{aligned}$$

From the elementary inequality $-y \le (\exp (-y)-1) \cdot (1+y/2)$ for $y \ge 0$ we conclude, taking $y = -\log \eta$, that $\Lambda _r < 0$. The same inequality applies also to the limits $\eta \rightarrow \{0^+,1^-\}$.

Proposition 3.1 provides a sufficient condition for stability based only on the size of the tumor and can be thought of as a modeling cut-off: either a radially symmetric tumor is small enough to be stable, or it has grown too large relative to the oxygen source for stability to be guaranteed for all $\eta$. While there might exist values of $\eta$ for which a larger tumor is stable, the size alone for $r_p^{\textrm{eq}}> \exp (-1)$ is not sufficient to deduce that the tumor is stable, but the converse is true for smaller tumors. In the numerical experiments in Sect. 4, we use Proposition 3.1 to ensure that the tumor is small enough that a radially symmetric solution is expected to reach a stable stationary state. For suggested stationary radii $(r_n^{\textrm{eq}},r_q^{\textrm{eq}},r_p^{\textrm{eq}})$, with $r_p^{\textrm{eq}}$ small enough, $\mu _{\textrm{death}}$ is defined by setting (3.4) to 0, and similarly $K_{\textrm{prol}}$ and $K_{\textrm{death}}$ are found from (3.3), which are used to determine the stability of the stationary state and the full dynamics of the tumor’s growth rate.

### Morphological Stability

We analyze the stability of the PDE model by studying the system’s response to perturbations of a radially symmetric solution. The main result depends on the three Lemmas in Sect. A and reads as follows:

#### Theorem 3.2

(Linear stability) Let the outer tumor boundary $r_{p}$ be perturbed by

3.6 $$\begin{aligned} \tilde{r}_{p}(\theta ) = r_{p} + \epsilon \alpha _k^{(p)}\cos (k\theta ), \end{aligned}$$

for some $|\epsilon |\ll 1$. Write the induced inner perturbations on the same form,

3.7 $$\begin{aligned} \begin{aligned} \tilde{r}_{q}(\theta )&= r_{q} + \epsilon \alpha _k^{(q)} \cos (k\theta ), \\ \tilde{r}_{n}(\theta )&= r_{n} + \epsilon \alpha _k^{(n)} \cos (k\theta ), \end{aligned} \end{aligned}$$

each defined as the interface between the regions of different cellular growth rates according to (2.10) with the oxygen field governed by (2.15) (see also Fig. 2). We let the pressure field and the cell density advection be defined as in Sect. 2.3. Then to first order in $\epsilon$, the *k*th perturbation mode in (3.6) grows as $\alpha _k^{(p)}(t) \propto e^{\Lambda (k) t}$ according to the dispersion relation

3.8 $$\begin{aligned} \Lambda (k)&= \frac{r_{p}'}{r_{p}}\Biggl ( \overbrace{\frac{1 - D_{\textrm{ext}}}{1+D_{\textrm{ext}}}k }^{\text {Saffman-Taylor}} - 1 \Biggr ) \\ \nonumber +&\frac{D_{\textrm{ext}}}{1+D_{\textrm{ext}}} \Biggl (1 - \underbrace{r_{p}^{-k -1}\left( \mu _{\textrm{death}}r_{n}^{k+1}\frac{\alpha _k^{(n)}}{\alpha _k^{(p)}} + r_{q}^{k+1}\frac{\alpha _k^{(q)}}{\alpha _k^{(p)}}\right) }_{\text {inner region perturbation}} - \underbrace{\sigma \frac{k(k^2-1)}{r_{p}^{3}}}_{\text {surface tension}}\Biggr ), \end{aligned}$$

in which the interface perturbation coefficients $\alpha _k^{(q)}$, $\alpha _k^{(n)}$ are given by (A.11) and where the radial growth follows from (3.4),

3.9 $$\begin{aligned} r_{p}'&= -\frac{1}{2r_{p}}(\mu _{\textrm{death}}r_{n}^2 + r_{q}^2 - r_{p}^{2}) = -p{'}(r_{p}), \end{aligned}$$

by Darcy’s law.

#### Proof

We first solve for the pressure field (2.9) under the assumption of radial symmetry where, again, the divergence theorem implies Neumann interface conditions. The result is

3.10 $$\begin{aligned} p^{(\textrm{ext})}(r)&= (\mu _{\textrm{death}}r_{n}^2 + r_{q}^2 - r_{p}^2) \frac{1}{2D_{\textrm{ext}}} \log (r),&\quad r_{p} \le r&, \end{aligned}$$

3.11 $$\begin{aligned} p^{(p)}(r)&= (\mu _{\textrm{death}}r_{n}^2 + r_{q}^2) \frac{1}{2} \log \frac{r}{r_{p}} - \frac{1}{4}(r^2 - r_{p}^{2}) + p^{(\textrm{ext})}(r_{p}) - \sigma C,&\quad r_{q} \le r&\le r_{p}, \end{aligned}$$

3.12 $$\begin{aligned} p^{(q)}(r)&= p^{(p)}(r_{q}) + \frac{1}{2}r_{n}^2\log \frac{r}{r_{q}},&\quad r_{n} \le r&\le r_{q} , \nonumber \\ p^{(n)}(r)&= p^{(q)}(r_{n}) + \frac{\mu _{\textrm{death}}}{4}(r^2 - r_{n}^2),&\quad 0 \le r&\le r_{n}, \end{aligned}$$

where the negative pressure gradient at $r_{p}$ (3.11) recovers the velocity of the tumor’s outer rim (3.4) as expected.

We continue by perturbing the tumor boundary according to (3.6) and, assuming independence between modes for the induced perturbations, we have (3.7) and let the pressure perturbation in each region $i \in \{\text {ext},p,q,n\}$ be

$$\begin{aligned} \tilde{p}^{(i)}(r)&= p^{(i)}(r) + \epsilon \gamma _k^{(i)}(r) \cos (k \theta ). \end{aligned}$$

Using similar arguments to those in the proof of Lemma A.3, we can show that the pressure perturbation coefficients are of the form (A.13). We next use Lemmas A.1 and A.2 to find the continuity relations for the coefficients. However, the pressure discontinuity at the tumor boundary, (2.13) and (2.14), must be treated separately. For the former, the first order approximation in $\epsilon$ of $\sigma C(r_{p}^*)$ is evaluated, and for the latter we use that *D* and $D_{\textrm{ext}}$ are constant within their respective domains. Thus, the continuity relations become

3.13 $$\begin{aligned} \begin{aligned} \gamma _k^{(p)}\left( r_{p}\right)&= \gamma _k^{(\textrm{ext})}(r_{p}) + (p^{(\textrm{ext})}{'}(r_{p}) - p{'}(r_{p}) + \sigma \frac{k^2 - 1}{r_{p}^{2}}) \alpha _k^{(p)}, \\ \gamma _k^{(p)}(r_{q})&= \gamma _k^{(q)}(r_{q}), \\ \gamma _k^{(q)}(r_{n})&= \gamma _k^{(n)}(r_{n}), \end{aligned} \end{aligned}$$

and for the derivatives,

3.14 $$\begin{aligned} \begin{aligned} \gamma _k^{(p)}{'}(r_{p})&= - p{''}(r_{p})\alpha _k^{(p)} + D_{\textrm{ext}}(\gamma _k^{(\textrm{ext})}{'}(r_{p}) + p^{(\textrm{ext})}{''}(r_{p})\alpha _k^{(p)}), \\ \gamma _k^{(p)}{'}(r_{q})&= \gamma _k^{(q)}{'}(r_{q}) + \alpha _k^{(q)}(p^{(p)}{''}(r_{q}) - p^{(q)}{''}(r_{q})), \\ \gamma _k^{(q)}{'}(r_{n})&= \gamma _k^{(n)}{'}(r_{n}) + \alpha _k^{(n)}(p^{(q)}{''}(r_{n}) - p^{(n)}{''}(r_{n})). \\ \end{aligned} \end{aligned}$$

As in Giverso et al. (2016), we find the form of this dispersion relation from the velocity at the tumor boundary by applying (2.11) to the perturbed solution. Considering only the first order terms in $\epsilon$, we find that $\partial \alpha _k^{(p)} / \partial t = \Lambda (k) \alpha _k^{(p)}$, with

3.15 $$\begin{aligned} \Lambda (k) = -\left( p{''}(r_{p}) + \frac{\gamma _k^{(p)}{'}(r_{p})}{\alpha _k^{(p)}}\right) . \end{aligned}$$

Finally, evaluating (3.15) using Lemma A.3 for the coefficients $\alpha _k^{(q)}$, $\alpha _k^{(n)}$ yields the dispersion relation (3.8).

Note that (3.8) is independent of the coefficients $\alpha _k^{(p)}$ of the initiating perturbation since both $\alpha _k^{(n)}$ and $\alpha _k^{(q)}$ are proportional to $\alpha _k^{(p)}$, as seen in (A.11). It follows that $\Lambda (k)$ is unambiguously determined by the set of parameters and values $\{r_{p}, r_{q}, r_{n}, \mu _{\textrm{death}}, D_{\textrm{ext}}, \sigma \}$.

The first part in (3.8) is the Saffman–Taylor instability (Saffman and Taylor 1958) term. This type of instability has previously been discussed in the context of growing cell populations (Mather et al. 2010). Further, the induced perturbations on the oxygen field act to dampen any morphological instability as seen by the inner region perturbation term, which is negative and can only decrease the value of $\Lambda (k)$. However, for large *k* this dampening vanishes in general as is seen from the following reasoning. Let $r_{n}^2 = \theta _n r_{p}^2$, $r_{q}^2 = \theta _q r_{p}^2$, with $0 \le \theta _n< \theta _q < 1$, i.e., the regions do not overlap. Then the inner region perturbation term becomes

3.16 $$\begin{aligned} \frac{1 - r_{p}^{2k}}{1 - \theta _n^k r_{p}^{2k}} \left( \mu _{\textrm{death}}\theta _n^{k} + \frac{\theta _q^{k} - \theta _n^{k}}{\theta _q - \theta _n} \times \frac{1}{k} \times \theta _q \right) , \end{aligned}$$

which for $r_{p} < 1$ tends to zero as *k* grows. Finally, surface tension also reduces the amplitude and range of unstable perturbation modes. Similar effects are observed due to cell adhesion in glioblastoma models in silico and in vitro (Oraiopoulou et al. 2018).

### Notable Special Cases

The dispersion relation (3.8) provides rich insight into the morphological dynamics of our model of a growing avascular tumor. We outline notable regimes of these dynamics below.

The Saffman–Taylor instability When the tumor grows in a medium that flows on a significantly smaller timescale than the migration rate of the tumor cells, corresponding to $D_{\textrm{ext}}\gg D$ ($\equiv 1$ by nondimensionalization), the dispersion relation becomes

3.17 $$\begin{aligned} \Lambda (k)&= -\frac{r_{p}'}{r_p}( k + 1 ) \\ \nonumber&+1 - r_p^{-k-1}\left( \mu _{\textrm{death}}r_n^{k+1}\frac{\alpha _k^{(n)}}{\alpha _k^{(p)}} + r_q^{k+1}\frac{\alpha _k^{(q)}}{\alpha _k^{(p)}}\right) - \sigma \frac{k(k^2-1)}{r_p^{3}}. \end{aligned}$$

The same result is obtained by assuming a homogeneous outer pressure, $p^{(\textrm{ext})}(r) = p_0$, for some constant $p_0$. Following the same arguments we get again (3.13) but with $\gamma _k^{(\textrm{ext})}$ and $p^{(\textrm{ext})}{'}$ equal to zero, and that (3.14) holds except for the first relation, since $C^1$-continuity is no longer valid on $\partial \Omega$ making Lemma A.2 inapplicable there. The dispersion relation is still given by (3.15) and Lemma A.3 also holds (the oxygen field does not explicitly depend on the outer pressure), which combined yields (3.17). The Saffman–Taylor term (the first term) is here at its most stabilizing since $k(1 - D_{\textrm{ext}})/(1 + D_{\textrm{ext}}) \ge -k$ for $D_{\textrm{ext}}\ge 0$. On the other side of the spectrum, we have the situation when the external tissue is significantly more viscous and practically immovable within the temporal scale of the growing tumor. This corresponds to the condition $D_{\textrm{ext}}\ll 1$, and (3.8) becomes

3.18 $$\begin{aligned} \Lambda (k)= &  \frac{r_{p}'}{r_p}( k - 1 ), \end{aligned}$$

and every mode $k \ge 2$ is unstable during growth with no stabilizing effect from the surface tension. The case $D_{\textrm{ext}}< 1$ is the common form of the Saffman–Taylor instability.

Growth phases The tumor’s morphological stability depends on which growth phase the tumor is in. We identify the following quantity as a discriminant of the tumor’s growth phase:

3.19 $$\begin{aligned} \Delta _{\theta } = \frac{r_{p}'}{r_{p}}, \end{aligned}$$

i.e., the relative tumor boundary velocity. When the tumor is initially growing in a nutrient-rich environment and $r_n$, $r_q$ are small, then from (3.9) we see that $r_{p}' \approx r_{p}/2$ which implies that $\Delta _{\theta } \approx \frac{1}{2}$ and hence (3.8) becomes

3.20 $$\begin{aligned} \Lambda (k) = \frac{1-D_{\textrm{ext}}}{2(1 + D_{\textrm{ext}})}(k-1) - \frac{D_{\textrm{ext}}}{1+D_{\textrm{ext}}} \sigma \frac{k(k^2-1)}{r_p^3}. \end{aligned}$$

Hence as the tumor grows exponentially, we expect all modes $k > 1$ to be stable for $D_{\textrm{ext}}> 1$ and unstable for $D_{\textrm{ext}}< 1$ in the absence of surface tension effects.

As the tumor grows larger and the nutrient availability can no longer sustain the entire tumor, the tumor front slows down and $\Delta _{\theta } \rightarrow 0^+$, and the effect from the Saffman–Taylor part diminishes. From (3.16) we see that the inner region perturbation term in (3.8) is negative, and hence close to the tumor’s stationary state we have that

3.21 $$\begin{aligned} \Lambda (k) < \frac{D_{\textrm{ext}}}{1+D_{\textrm{ext}}} \Bigl (1 - \sigma \frac{k(k^2-1)}{r_{p}^{3}}\Bigr ), \end{aligned}$$

Since the inner region perturbation term tends to zero for increasing *k*, the upper bound becomes a good approximation of $\Lambda (k)$ for large *k*. Clearly, (3.21) shows that a positive value of $\sigma$ is necessary for the stationary stability.

Surface tension and stationarity The stability relation provides an estimate of the surface tension required to maintain a radially symmetric growth as $t \rightarrow \infty$. Considering only the case when $D_{\textrm{ext}}\gg 1$, we see from (3.17) that the least stable case with respect to the discriminant is obtained when $\Delta _{\theta } = 0$. We find the necessary surface tension by requiring $\Lambda (k) = 0$ for all *k*. Again, using that the inner region perturbation term is negative, we obtain from (3.17) the bound

3.22 $$\begin{aligned} \sigma _{\text {stable},k} \le \frac{r_p^{3}}{k(k^2 - 1)} \end{aligned}$$

where $\sigma _{\text {stable},k}$ is the lower bound of $\sigma$ required for stabilizing small perturbations of mode *k*. Thus, to stabilize all modes $k \ge 2$ it is *sufficient* to have that $\sigma = r_p^{3}/6$, depending only on the total tumor volume. Again, since the inner perturbation term tends to zero as *k* grows, the upper bound is a good approximation of $\sigma _{\text {stable},k}$ for large *k*.

Creeping instability We finally remark on the interesting mode $k=1$, the only mode unaffected by surface tension. Geometrically, this mode corresponds to movement of the tumor’s center of mass: the tumor begins to *creep* towards the oxygen source given a small perturbation. From (3.8),

3.23 $$\begin{aligned} \Lambda (k=1) = \frac{D_{\textrm{ext}}}{1 + D_{\textrm{ext}}}\frac{\mu _{\textrm{death}}r_n^2 + r_q^2}{r_p^2} \times \frac{r_p^2 - r_n^2}{1-r_n^2} \ge 0. \end{aligned}$$

Thus, the tendency for creeping always exists when $r_{q}$ and/or $r_{n}$ are positive, and the model would require additional features regarding, e.g., the external tissue’s response to invasion, in order to inhibit this effect. Note that this tendency is reduced for tumors growing within an environment more viscous and/or less permeable than its own. As showed previously, however, such conditions make all the other modes $k\ge 2$ *less* stable.

## Numerical Examples

In the following section, we present numerical simulations of both the stochastic model from Sect. 2.2 and the PDE model from Sect. 2.3. We focus on the case $D_{\textrm{ext}}\gg 1$ which we showed had an inherent stabilizing effect during growth in Sect. 3.3. In Sect. 4.1 we assess the validity of the assumption that growth is radially symmetric and how the stability responds to surface tension. In Sect. 4.2, we explore the relation between surface tension and the emergent morphology and compare the outcomes between the stochastic and the mean-field PDE model.

Due to certain differences between the models, we use effective parameter values for the PDE simulations for the parameters $\mu _{\textrm{prol}}$, $\mu _{\textrm{death}}$ and $\lambda$, and denote those with a bar, e.g., $\bar{\mu }_{\textrm{prol}}$. The effective parameters are derived from the stochastic model simulations via basic scaling considerations or preliminary simulations as detailed in Sect. B.

The set of parameters used in both the DLCM and the PDE simulations are found in Table 2.

**Table 2:** Standard set of parameters of the DLCM model and the corresponding effective PDE parameters

Parameter	DLCM	PDE
$\mu _{\textrm{death}}$	0.5	1.35
$\mu _{\textrm{deg}}$	0.05	N/A
$\kappa _{\textrm{prol}}$	0.94	0.94
$\kappa _{\textrm{death}}$	0.93	0.93
$\lambda$	1	1.15
$p^{(\textrm{ext})}$	0	0
$D_{\textrm{ext}}$	$+ \infty$	$+ \infty$
$D_2$	25	N/A

### Radial Symmetry and Surface Tension

We first solve the PDE under the assumption of radial symmetry. We solve the reduced 1D problem comprising (3.3) and (3.4) as derived in Sect. 3.1 and evaluate the perturbation growth rates (3.17) during tumor growth and study their response to surface tension. For quantitative measurements of the regional characteristics during tumor growth, we consider the volumetric quantities $V_p = \pi r_p^2$, $V_q = \pi r_q^2$, and $V_n = \pi r_n^2$.

**Fig. 3:**
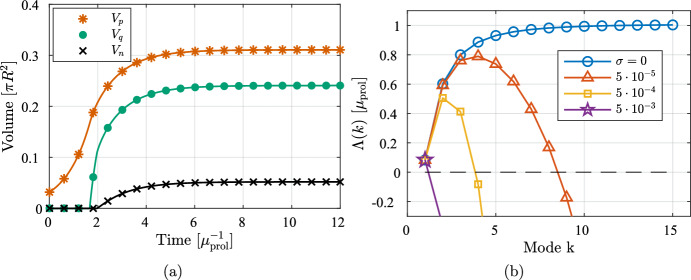
Regional characteristics and perturbation growth rates under the assumption of radial symmetry. **a** The evolution of the three characteristic regions. **b** The perturbation growth rates (3.17) for the first few modes at the tumor’s stationary state and at varying surface tension $\sigma$

Figure 3 shows the evolution of the regional characteristics for a simulation using the standard parameters for the PDE found in Table 2, accompanied by the perturbation growth rates (3.17) at the stationary state. In Fig. 3a we observe the emergence of the characteristic sigmoidal growth of the total volume, with an initially exponential growth followed by a growth rate that plateaus. Figure 3b shows the perturbation growth rates versus mode close to the stationary state for a range of $\sigma$. It is clear that the assumption of radial symmetry for low values of $\sigma$ does not hold when oxygen is not sufficient to sustain the growth of the entire population. From (3.17) we find that $\sigma _{\text {stable},2} \approx 3.1\cdot 10^{-3}$, i.e., this is the value needed to stabilize modes $k \ge 2$. From Fig. 3b we see that $\sigma$ lower than this prompt nontrivial spatial behavior with instabilities that may occur over different timescales (investigated further in Sect. 4.2).

**Fig. 4:**
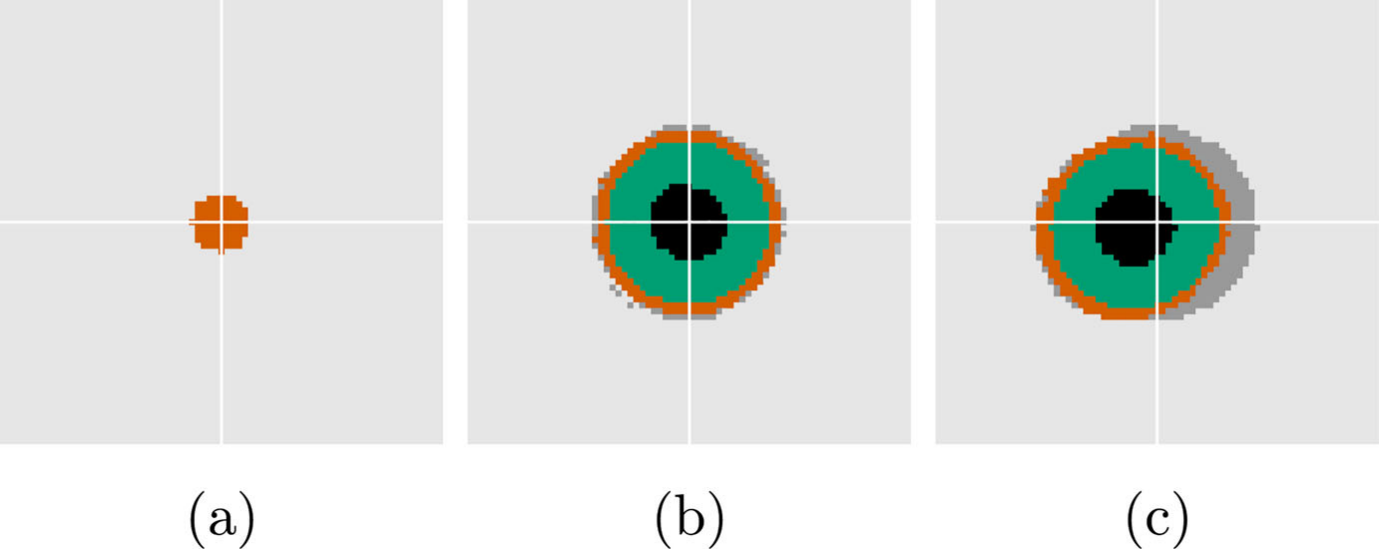
Solution using a surface tension large enough to ensure stability and hence radial symmetry, $\sigma = 3.2\cdot 10^{-3}$. Times $t=0$, $t=10$, $t=80$, respectively, from left to right. *Red:* proliferative cells, *green:* quiescent cells, *black:* necrotic cells, *dark grey:* previously occupied. The white cross-hairs are centered on the domain origin

As suggested by (3.23), creeping is expected for long enough times. We test this by simulating the model using $\sigma = 3.2\cdot 10^{-3}$ to ensure that modes $k \ge 2$ are stabilized (see details about the numerical methods and the implementation of surface tension in Sect. B). The results are shown in Fig. 4 where we see that the tumor reaches close to its stationary state at around $t = 10$, in accordance with the solution to the 1D equations in Fig. 3. We observe a notable collective migration from the domain origin starting at $t = 80$.

### The Emergent Morphology and its Response to Stochasticity

**Fig. 5:**
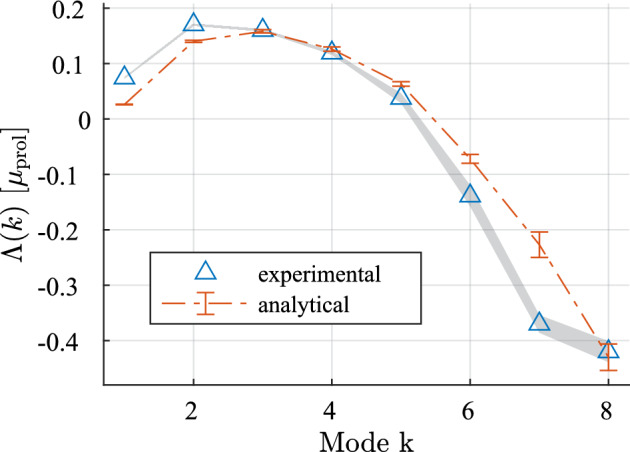
Comparison between analytical (3.17) and experimental perturbation growth rates for the first few modes when $\sigma = 10^{-4}$. The error bars show the standard deviation of the analytical values during the time interval of measurement, and the shaded region corresponds to the standard deviation of the growth rate estimate during the same interval (20 independent runs)

We begin by a brief investigation into how well the dispersion relation (3.17) describes the morphological stability of the DLCM model tumor. To this end, we conduct simulations of the complete DLCM model, initialized close to the estimated equilibrium volumes (see Fig. 3b) using the standard parameters and setting $\sigma = 10^{-4}$. We impose an initial perturbation to the tumor geometry of the form (3.6) with $\epsilon = 0.05$ and for modes $k = 1,..., 8$. We regard the mean of (3.17) during a selected time interval as an analytical prediction and measure the growth of each mode by fitting the amplitude to an exponential growth law. Figure 5 indicates that the rates agree fairly well, thus supporting the use of the PDE-based stability analysis in predicting the behavior also of the DLCM model.

Motivated by the quantitative evidence for a correspondence between the PDE analysis and the DLCM model, we compare the morphology and the growth patterns of the stochastic model and the PDE model for similar parameters. To avoid perturbations that are biased by the discretization method we add small amounts of white noise to the cell density updates. These and further details of the PDE solver, including the implementation of surface tension, are discussed in Sect. B. We evaluate the tumor boundary *roundness* defined over a 2D region as

4.1 $$\begin{aligned} \text {Roundness} = \frac{4\pi \times \text {Area}}{\text {Perimeter}^2}, \end{aligned}$$

which ranges from 0 to 1, where 1 means the shape is perfectly circular and smaller values measure its deviation from circularity.

We first investigate numerically the effects of surface tension on the tumor morphology. Specifically, we study the onset of second mode instability according to (3.17) for a tumor growing in two spatial dimensions. For this purpose, we compare the growth using $\sigma = 2 \cdot 10^{-3}$ and $\sigma = 5 \cdot 10^{-4}$ until $t = 30$, during which the former value is stable for $k=2$ although it is not stable over larger times *t*. For both experiments, we compare morphology and growth using a single simulation per model (a discussion on the impact of stochasticity is offered in Sect. 5).

**Fig. 6:**
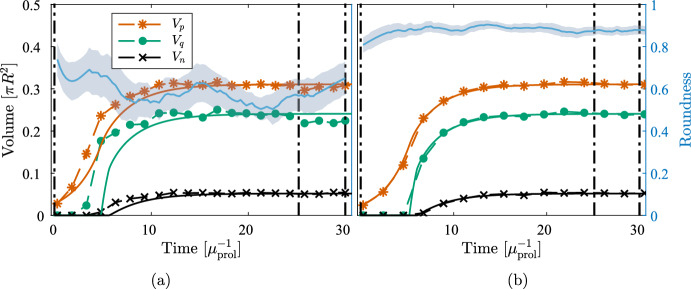
Evolution of characteristic volumes for DLCM (**a**) and PDE (**b**), respectively, using the standard parameters in Table 2 and $\sigma = 2 \cdot 10^{-3}$. The 2D solutions (dashed) are compared with the 1D solution (3.3)–(3.4) (solid) for the same parameters. Blue shows the roundness (4.1) of the tumor boundary over time with moving window standard deviation in shaded. The vertical lines indicate the times $t\in [0,25,30]$ of the solutions shown in Fig. 7

**Fig. 7:**
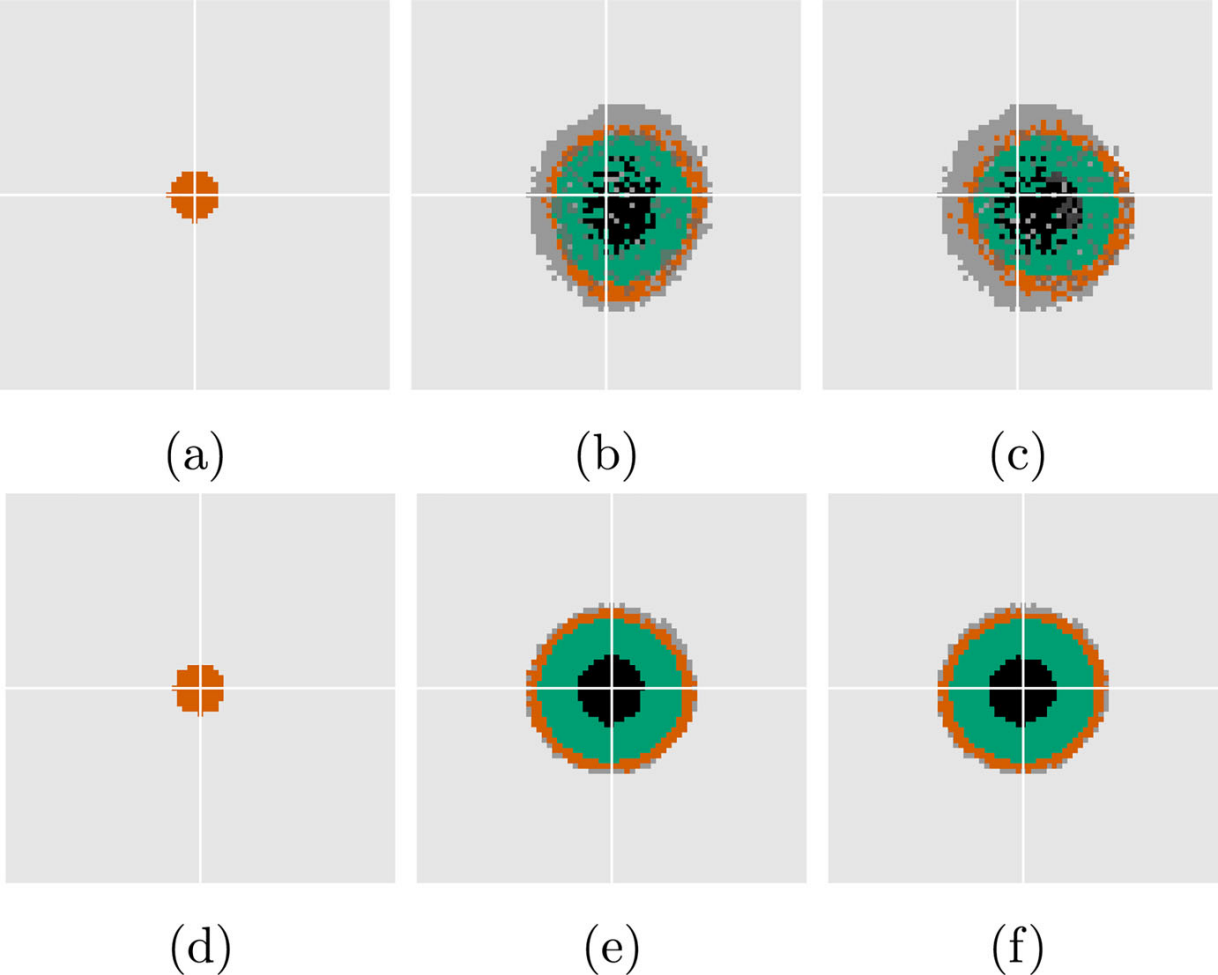
Solutions corresponding to Fig. 6 at $t = 0$, 25, 30, respectively, with DLCM in the top row and the PDE in the bottom row. Color scheme as in Fig. 4. For DLCM, darker gray shows quiescent cells below $\kappa _{\textrm{death}}$, and darker shades of red and green indicate doubly occupied voxels

Figure 6 shows the evolution of the tumor’s characteristic volumes for both models together with the tumor roundness (4.1) using the larger value of $\sigma$. Figure 7 shows snapshots of the solution corresponding to Fig. 6. Both solutions remain close to being radially symmetric during the full simulations since the small second mode instability does not show during these relatively short time intervals. Notably, the creeping effect becomes apparent earlier for the DLCM simulations.

**Fig. 8:**
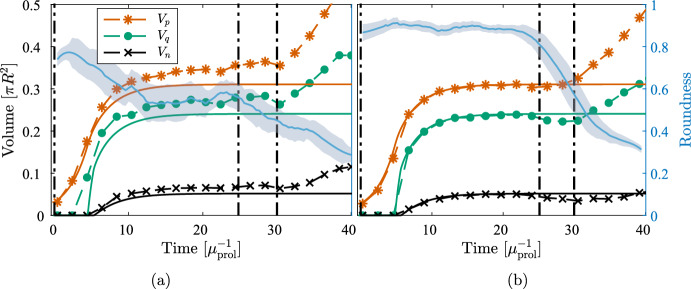
Solutions from DLCM (left) and PDE (right), respectively, using the standard parameters Table 2 and $\sigma = 5 \cdot 10^{-4}$, which is insufficient to ensure radial symmetry. The 2D solutions (dashed) are compared with the 1D solution (3.3)–(3.4) (solid) using the same parameters and shown in the same units of time. The PDE model tumor splits into two parts just before the final time where the roundness metric is undefined. The vertical lines indicate the times $t \in [0, 25, 30]$ of the spatial solutions shown in Fig. 9

**Fig. 9:**
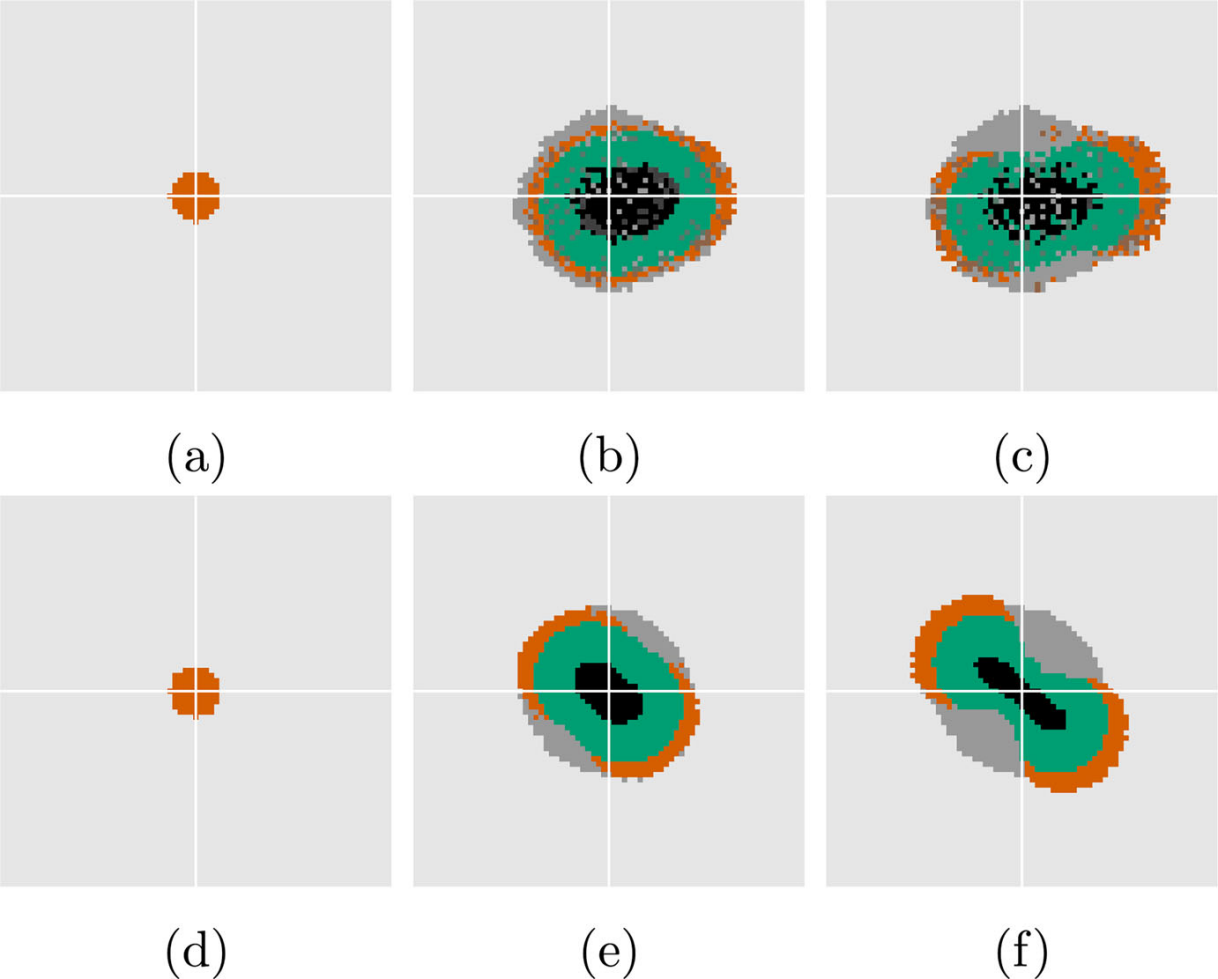
Solutions corresponding to Fig. 8 at $t = 0$, 25, 30, respectively. Color scheme as in Fig. 7

Similarly, Figs. 8 and 9 shows regional evolution and spatial solutions, respectively, for the lower value of $\sigma$. Both models display a significant decrease in roundness (accompanied by a total volume increase) as the tumor begins to split in two some time after the growth has plateaued. A notable difference between the growth curves during this process is that the DLCM tumor does not reach a fully stationary state before the splitting, possibly due to the higher exposure to noise in the stochastic model.

**Fig. 10:**
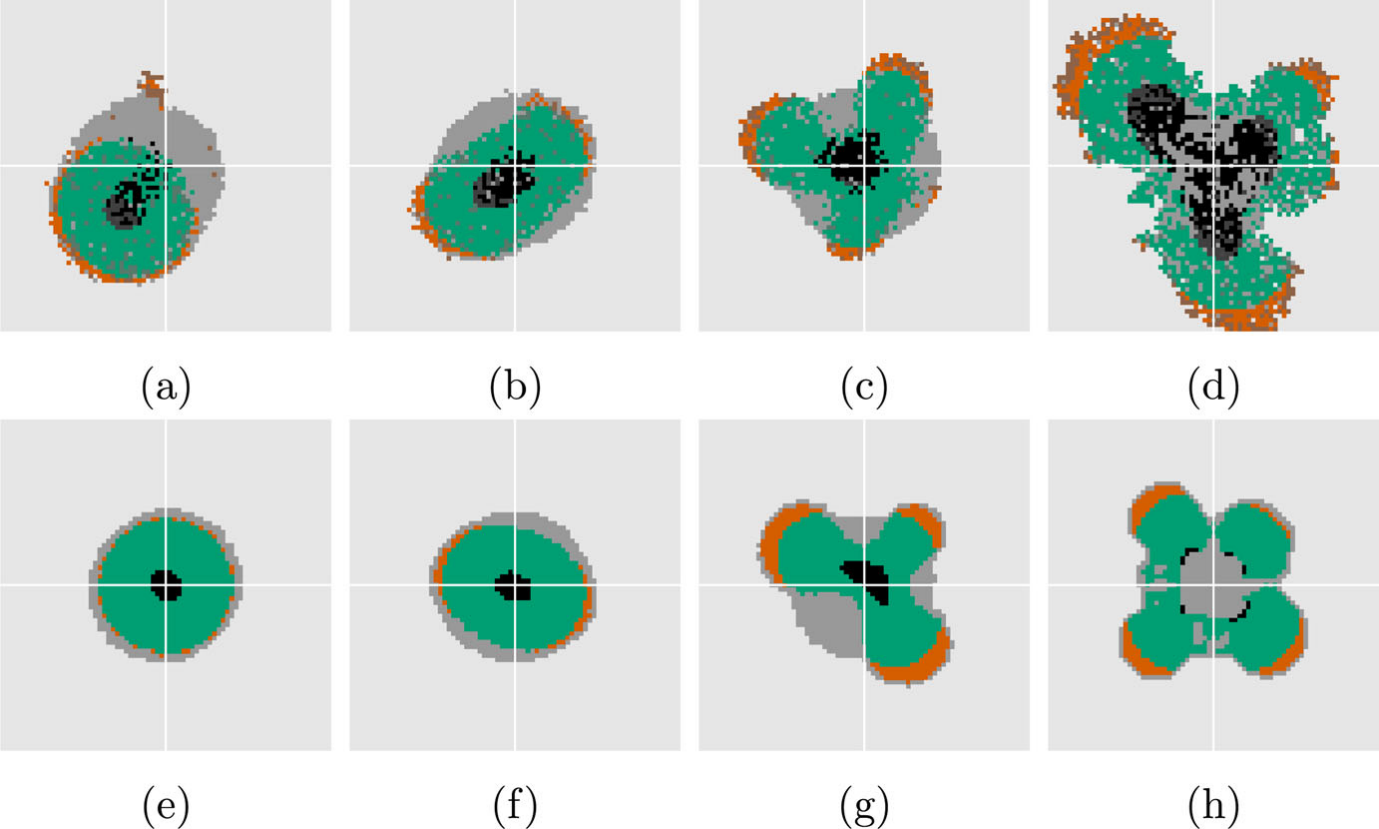
Solutions to DLCM (top row) and the PDE (bottom row) for various parameters highlighting different aspects of the model in relation to (3.8). Left to right shows decreasing surface tension with $\sigma = 2 \cdot 10^{-3}$, $7 \cdot 10^{-4}$, $1 \cdot 10^{-4}$, 0, and top row uses parameters $\kappa _{\textrm{prol}}= 0.94$, $\kappa _{\textrm{death}}= 0.92$, $\mu _{\textrm{death}}= 0.5$, with bottom row using effective parameters $\bar{\mu }_{\textrm{death}}= 1.0$, $\bar{\lambda }= 1.1$. Solutions are shown at $t = 30$. Color scheme as in Fig. 7

Finally, Fig. 10 shows the resulting morphology of both models using four different and smaller values of $\sigma$. For these experiments we use use a lower $\kappa _{\textrm{death}}= 0.92$ for a thinner proliferating rim which results in effective parameters $\bar{\mu }_{\textrm{death}}= 1.0$ and $\bar{\lambda }= 1.1$. Again, the radially symmetric tumor displays significant creeping only for the DLCM model at the selected final time (cf. Fig. 10a, e). The morphologies of the tumors are similar in terms of emergent unstable modes and sizes of the characteristic regions, e.g., the tumors beginning to separate into two is seen in both Fig. 10b, f. For the most unstable case using $\sigma = 0$ in Fig. 10d, the DLCM tumor grows somewhat larger and with different morphological and regional characteristics. Finally, Fig. 10a, c display small cell clusters detaching from the tumor to grow on their own, a phenomena that we never observe in our simulations of the PDE model (cf. Fig. 10e, g).

## Discussion

We have analyzed the morphological stability of a PDE model of avascular tumors. The PDE was derived to closely represent the mean-field of a stochastic model expressed in the DLCM framework. Assuming radial symmetry in the PDE model, we first characterized the growth dynamics as well as the stationary state. A linear stability analysis in two dimensions was subsequently carried out and we found a dispersion relation that describes the stability of the morphology of the tumor. Finally, we compared the analytical predictions with numerical simulations of both the stochastic DLCM and the PDE model. The observed morphology of the stochastic model was found to be in line with the predictions from the PDE analysis, including also the proposed relations required for a stable stationary state.

The Saffman–Taylor instability acts on the tumor boundary, where the determining factors are the porous medium permeability and the tissue viscosity as summarized in the coefficients *D* and $D_{\textrm{ext}}$. For a transient tumor growth, these coefficients determine whether the tumor boundary is stable ($D_{\textrm{ext}}> D$) or unstable ($D_{\textrm{ext}}< D)$. In the former case, as the tumor growth slows down due to oxygen starvation, perturbations on the boundary are amplified, thus destroying radial symmetry unless the surface tension parameter $\sigma$ is large enough; this was shown analytically in Sect. 3.3 and experimentally in Sect. 4.1. This also explains the asymmetry and unlimited growth of the original DLCM tumors presented in Engblom et al. (2018), which did not implement an explicit surface tension effect.

Morphological instability arising in conditions when nutrients are scarce is in line with analyses and simulations of other models of tumor growth and cell colonies. The model in Cristini et al. (2003) is a fluid-based PDE model including Darcy flow, with cell growth proportional to nutrient level, and a constant apoptosis rate. The model boundary velocity is explicitly dependent on the nutrient gradient, in contrast to the model analyzed herein that nevertheless displays a similar nutrient dependent growth instability. Further examples show similar instabilities in nutrient-deprived conditions both in agent-based models of cell colonies (Gerlee and Anderson 2007) and in hybrid models of tumor growth including cell cycles and ECM (Anderson et al. 2006). The former model tumor expands solely due to cell proliferation and not to pressure-driven migration, thus suggesting the persistence of this type of instability across a range of models.

Interestingly, our model predicts a creeping effect in which even an otherwise stable tumor as a whole migrates towards the oxygen source. Experiments in Sect. 4.2 suggest that the stochastic model has a somewhat stronger tendency for creeping than the PDE model. The larger noise levels of the DLCM model is a good candidate explanation for this difference. Moreover, the creeping effect cannot be inhibited by surface tension, but must be controlled through additional mechanisms of the model such as an elastic response from the external tissue (see Walker et al. 2023 for a review on minimal morphoelastic tumor models). Thus, the creeping phenomena prompts the following questions: Does creeping occur in vitro or in vivo and if so, over what timescales? If not, what mechanisms keep it from occurring; alternatively, what assumptions could make our model more realistic in this regard?

An additional observation from the DLCM model was the detachment of cell clusters even in the case of a surface tension large enough to support a radially symmetric solution. This is most likely due to the discrete and random nature of individual cells in the model. Detachment is therefore a distinct feature of on-lattice stochastic modeling in this context, which appears to be a more realistic representation of tumors growing under noisy conditions than a purely fluid mechanical continuous model can offer. That said, the stochasticity of the DLCM model does not have a significant impact on the region volumes and final size of a radially symmetric tumor. Rather, assuming stability, these outcomes are fairly accurately governed by the deterministic relations (3.3)–(3.4). The process noise does, however, have an impact on the morphology of the tumor under less surface tension, which implicitly affects the tumor size first when the boundary has been significantly distorted. A deeper investigation of this is outside the scope of the experiments reported here.

One fundamental difference between the two models is the spatial exclusion principle which is implemented in the DLCM framework via the carrying capacity. A consequence of this can be seen when comparing Fig. 9c, where the PDE tumor is close to separating into two pieces, while the DLCM tumor in contrast retains an oval shape. The latter is due to necrotic cells which degrade while still occupying voxels, thereby slowing down the mass flow. Similarly, Fig. 10d, h display significant differences in morphology and size, where the former model supports a larger necrotic region. These examples highlight emergent differences between the two ways of modeling cell extent and migration and call for an input of biological observations to approach a higher level of realism.

We end by briefly mentioning some potential modifications that may improve on the expressive power of the model. Considering the PDE model first, we see from (3.11) that the ambient pressure becomes very large for a stiff external medium. Thus, when modeling such scenarios the addition of pressure-dependent effects such as pressure-driven oxygen flow or a pressure-based proliferation rate become relevant. Such additions carry over to the stochastic framework in a fairly straightforward manner. To further improve on the realism of the nutrient modeling, a limit on the diffusion flux of the oxygen into the tumor across its boundary could also be considered (cf. Folkman and Hochberg (1973)). While these model modifications are readily implemented, the resulting emergent behavior is not obvious and a precise mathematical analysis is more involved due to the increase of nonlinear feedback mechanisms.

In conclusion, our basic stochastic avascular tumor model turned out to be a fruitful tool in leading to some new insights as well as investigations of the effects of various coupling- and feedback relations. Building and analyzing the mean-field PDE in tandem with the discrete stochastic model forced us to think more deeply in terms of trade-offs for continuum models of discrete cellular agents; this approach limits model refinements to a certain extent since the discrete and the continuous versions need to be consistent. We anticipate that the combination of a mean-field PDE and a stochastic model built from first principles will enable the development of filtering tools aimed specifically at integrative Bayesian approaches to data-driven applications. For example, the stochastic model can be used to characterize the process noise of a Kalman filter that leverages the PDE model as its state transition.

### Availability and Reproducibility

The computational results can be reproduced with release 1.4 of the URDME open-source simulation framework (Drawert et al. 2012), available for download at www.urdme.org (see the avascular tumor examples and the associated README in the DLCM workflow).

## Supporting Perturbation Results

We consider a continuous quantity *q* on two different circular domains $\Omega _1, \Omega _2 \subseteq \mathbb {R}^2$, where $\Omega _2$ is a circle of radius *r* inside $\Omega _1$, both domains sharing origin. We write $q^{(1)} = q|_{\Omega _1}$ and $q^{(2)} = q|_{\Omega _2}$. We assume *q* to be continuous across the closed interface between the two domains and that *q* is radially symmetric. We then introduce a small perturbation $\tilde{r}$ of *r*, and thus $\tilde{r} = \tilde{r} (\theta )$ for $\theta \in (-\pi ,\pi ]$, possibly inducing $q = q(s, \theta )$ when *q* depends on $\tilde{r}$ in some manner. Specifically, we let *q* depend on $\tilde{r}$ via the continuity properties of *q* across the interface and study how *q* responds to the perturbation in *r*.

### Lemma A.1

(Perturbation continuity) Let the interface be perturbed as

A.1 $$\begin{aligned} \tilde{r} (\theta )&= r + \epsilon \zeta _k \cos (k\theta ), \end{aligned}$$

for modes $$k = 1, 2,..., $$ and write the induced perturbation on *q* in the form

A.2 $$\begin{aligned} \tilde{q}(s,\theta )&= q(s) + \epsilon \phi _k(s) \cos (k\theta ), \end{aligned}$$

where *s* is some radial position in $\Omega _1 \cup \Omega _2$. We assume that the quantity *q* is continuous across the interface. Then, to first order in $\epsilon$,

A.3 $$\begin{aligned} q^{(1)}{'}(r)\zeta _k + \phi _k^{(1)}(r) = q^{(2)}{'} (r)\zeta _k + \phi _k^{(2)}(r), \quad k \ge 1, \end{aligned}$$

where $\phi _k^{(1)} = \phi _k|_{\Omega _1}$ and $\phi _k^{(2)} = \phi _k|_{\Omega _2}$, which is thus continuous across *r* if *q* is $C^1$ continuous.

### Proof

The quantities $\tilde{q}^{(1)}$ and $\tilde{q}^{(2)}$ are continuous across $\tilde{r}$, i.e.,

A.4 $$\begin{aligned} \tilde{q}^{(1)}(\tilde{r} ) = \tilde{q}^{(2)}(\tilde{r} ). \end{aligned}$$

We expand (A.4) according to the assumed structure of the perturbations (A.1) and (A.2) to get that

A.5 $$\begin{aligned} q^{(1)}(r) + \epsilon q^{(1)}{'}(r) \zeta _k \cos (k\theta )+ &  \epsilon \phi _k^{(1)}(r) \cos (k\theta ) + \mathcal {O}\left( \epsilon ^2\right) = \nonumber \\ q^{(2)}(r) + \epsilon q^{(2)}{'}(r) \zeta _k \cos (k\theta )+ &  \epsilon \phi _k^{(2)}(r) \cos (k\theta ) + \mathcal {O}\left( \epsilon ^2\right) . \end{aligned}$$

Now, the unperturbed quantities are assumed to be continuous across the unperturbed interface, i.e., $q^{(1)}(r) = q^{(2)}(r)$, and we arrive at (A.3) to first order in $\epsilon$.

Lemma A.1 extends in the obvious way in case there are multiple convex interfaces and the perturbation is imposed on one of them.

We next show how the derivatives of the perturbation coefficients relate.

### Lemma A.2

(Perturbation $C^1$-continuity) Under the same conditions as in Lemma A.1 and adding the assumption that $C^1$-continuity for *q* also holds across the interface, then to first order in $\epsilon$

A.6 $$\begin{aligned} q^{(1)}{''}(r)\zeta _k+ \phi _k^{(1)}{'}(r) = q^{(2)}{''} (r)\zeta _k + \phi _k^{(2)}{'}(r), \quad k \ge 1. \end{aligned}$$

### Proof

The directional derivative of a perturbed quantity in polar coordinates at any point on the interface $\tilde{r} (\theta )$ becomes

A.7 $$\begin{aligned} (\hat{n}_r, \hat{n}_{\theta }) \cdot \nabla \left( q(\tilde{r} ) + \epsilon \phi _k(\tilde{r} ) \cos (k\theta )\right) = \nonumber \\ q'(\tilde{r} )\hat{n}_s + \epsilon \phi _k'(\tilde{r} ) \cos (k\theta ))\hat{n}_s - \frac{k}{\tilde{r} }\epsilon \phi _k(\tilde{r} ) \sin (k\theta )\hat{n}_{\theta }, \end{aligned}$$

where $\varvec{n} = (\hat{n}_r, \hat{n}_{\theta })$ is the normal vector to $\tilde{r}$ in polar coordinates. The angular component, $\hat{n}_{\theta }$, is proportional to $\epsilon$ and thus the third term in (A.7) is $\mathcal {O}\left( \epsilon ^2\right)$. We use this simplified expression of the directional derivative for the $C^1$-continuity of $q^{(1)}$ and $q^{(2)}$ at $\tilde{r}$, and expand both around *r* to get

A.8 $$\begin{aligned} q^{(1)}{'}(r) + \epsilon q^{(1)}{''}(r) \zeta _k \cos (k\theta ) + \epsilon \phi _k^{(1)}{'}(r) \cos (k\theta ) + \mathcal {O}\left( \epsilon ^2\right) = \nonumber \\ q^{(2)}{'}(r) + \epsilon q^{(2)}{''}(r) \zeta _k \cos (k\theta ) + \epsilon \phi _k^{(2)}{'}(r) \cos (k\theta ) + \mathcal {O}\left( \epsilon ^2\right) . \end{aligned}$$

The derivatives are equal in the unperturbed case and again considering first order in $\epsilon$, we finally arrive at (A.6) above.

These two lemmas are enough to derive the propagation of a known perturbation on one interface through a system of $C^0$- and $C^1$-continuous quantities connected at shared interfaces. We continue by applying these lemmas to a perturbed oxygen field governed by (2.15) with three interfaces.

Preliminary perturbation response The pressure field (2.9) is coupled to the oxygen distribution obeying (2.15). To analyze the fully coupled system as is done in Sect. 3.2, an estimate of the response of an outer boundary perturbation into the inner regions via this feedback relation is required. As in Sect. 3.1 we assume a circular tumor with cell density $\rho$ approximately constant and equal to one, and hence the oxygen field *c* is initially given by (3.2). Let $\Omega _{\textrm{ext}}$, $\Omega _p$, $\Omega _q$, $\Omega _n$, $\subseteq$ $\mathbb {R}^2$, where $\Omega _n$ is a disk and the rest are annuli according to Fig. 2. The outer domain $\Omega _{\textrm{ext}}$ is the annulus with small radius $r_p$ and large radius *R*. We write $c^{(i)} = c|_{\Omega _i}$ for $i \in \{\text {ext},p,q,n\}$. The perturbations of the inner regions as induced from an arbitrary perturbation mode on $r_p$ and the continuity properties of *c* across each interface are then covered by the following result.

### Lemma A.3

(Perturbation response) Let the outer tumor boundary $r_{p}$ be perturbed by

A.9 $$\begin{aligned} \tilde{r}_{p}(\theta ) = r_{p} + \epsilon \alpha _k^{(p)}\cos (k\theta ), \end{aligned}$$

for some $|\epsilon |\ll 1$. Write the induced inner perturbations on the same form

A.10 $$\begin{aligned} \begin{aligned} \tilde{r}_{q}(\theta )&= r_{q} + \epsilon \alpha _k^{(q)} \cos (k\theta ), \\ \tilde{r}_{n}(\theta )&= r_{n} + \epsilon \alpha _k^{(n)} \cos (k\theta ), \end{aligned} \end{aligned}$$

each defined as the interface between the regions of different cellular growth rates according to (2.10) with the oxygen field governed by (2.15). Then the inner perturbations are to first order in $\epsilon$ given by

A.11 $$\begin{aligned} \begin{aligned} \alpha _k^{(q)}&= \frac{1}{k r_{q}^{k-1} r_{p}^{k-1}} \times \frac{r_{q}^{2k} - r_{n}^{2k}}{r_{q}^2 - r_{n}^2} \times \frac{1 - r_{p}^{2k}}{1-r_{n}^{2k}} \times \alpha _k^{(p)}, \\ \alpha _k^{(n)}&= \frac{r_{n}^{k-1}}{r_{p}^{k-1}} \times \frac{1 - r_{p}^{2k}}{1 - r_{n}^{2k}} \times \alpha _k^{(p)}. \end{aligned} \end{aligned}$$

### Proof

Similar to (A.10), we write the induced perturbation on the quantity (here the oxygen *c*) in each region $i \in \{\text {ext},p,q,n\}$ as

A.12 $$\begin{aligned} \tilde{c}^{(i)}(r,\theta ) = c^{(i)}(r) + \epsilon \beta _k^{(i)}(r) \cos (k\theta ). \end{aligned}$$

We find $\beta _k^{(i)}(r)$ by inserting (A.12) into the oxygen equation (2.15), using that the oxygen consumption is constant in each region, and considering only the first order terms in $\epsilon$:

A.13 $$\begin{aligned} \beta _k^{(i)}(r) = a_1^{(i)} r^k + a_2^{(i)} r^{-k}, \end{aligned}$$

for some constants $a_1^{(i)}$ and $a_2^{(i)}$. Assuming regularity at the origin we conclude that $a_2^{(n)} = 0$. The rest of the constants are determined from the continuity properties at the interfaces as follows.

Firstly, the oxygen is $C^1$-continuous across each interface, i.e.,

$$\begin{aligned} \tilde{c}^{(\textrm{ext})}(r_{p}) = c^{(p)}(r_{p}), \quad c^{(p)}(r_{q}) = c^{(q)}(r_{q}), \quad c^{(q)}(r_{n}) = c^{(n)}(r_{n}). \end{aligned}$$

and we may apply Lemma A.1 and A.2 to get that

A.14 $$\begin{aligned} \beta _k^{(\text {ext})}(r_{p}) = \beta _k^{(p)}(r_{p}), \quad \beta _k^{(p)}(r_{q}) = \beta _k^{(q)}(r_{q}), \quad \beta _k^{(q)}(r_{n}) = \beta _k^{(n)}(r_{n}), \end{aligned}$$

and

A.15 $$\begin{aligned} \begin{aligned} \beta _k^{(\textrm{ext})}{'}(r_{p}) - \beta _k^{(p)}{'}(r_{p})&= \lambda \alpha _k^{(p)}, \\ \beta _k^{(p)}{'}(r_{q}) - \beta _k^{(q)}{'}(r_{q})&= 0, \\ \beta _k^{(q)}{'}(r_{n}) - \beta _k^{(n)}{'}(r_{n})&= - \lambda \alpha _k^{(n)}, \end{aligned} \end{aligned}$$

where the right-hand sides were obtained by differentiation of the radially symmetric oxygen field, as given by (3.2).

Secondly, the oxygen levels at the inner interfaces are known and must equal the corresponding thresholds:

A.16 $$\begin{aligned} \tilde{c}^{(q)}(r_{n}) = \kappa _{\textrm{death}}, \quad \tilde{c}^{(q)}(r_{q}) = \kappa _{\textrm{prol}}. \end{aligned}$$

To each of (A.16) we now apply Lemma A.1. Considering again the first order terms, we get

A.17 $$\begin{aligned} \begin{aligned} \alpha _k^{(q)} c^{(q)}{'}(r_{q}) + \beta _k^{(q)}(r_{q})&= 0, \\ \alpha _k^{(n)} c^{(q)}{'}(r_{n}) + \beta _k^{(q)}(r_{n})&= 0. \end{aligned} \end{aligned}$$

Finally, combining (A.15), (3.2), and (A.17), we arrive at the response (A.11).

In summary, (A.11) specifies how the tumor’s inner interfaces $r_{q}$ and $r_{n}$ respond to an outer perturbation on $r_{p}$ given that the interfaces are defined by an oxygen field (2.15).

## Numerical Methods

PDE solution The pressure and oxygen fields (2.9) and (2.15), respectively, are readily solved by a finite element method (FEM). We solve for each quantity using linear basis functions and, for simplicity, using a lumped mass matrix. Solving for the oxygen, however, requires special considerations as the oxygen consumption depends on the oxygen level at each time step. To address this, we solve for the oxygen iteratively within a smaller time scale, denoted $\tau$, while holding $t_n$ and all other variables that depend on $t_n$ constant. We solve the resulting time-dependent heat equation implicitly using the Euler backward method, while treating the source-term explicitly. We iterate in pseudo-time $\tau$ until $\vert c^{\tau +1}-c^{\tau } \vert$ is less than some tolerance after which we set $c(t_{n+1}) = c^{\tau +1}$. The inner iterations are given by

B.1 $$\begin{aligned} \frac{c^{\tau +1} - c^{\tau }}{\Delta \tau } - \Delta c^{\tau +1} = {\left\{ \begin{array}{ll} - \lambda \rho (t_n), & c^{\tau } \ge \kappa _{prol} \text { on } \Omega _h(t_n) \\ 0, & \text {otherwise,} \end{array}\right. } \end{aligned}$$

where $\Omega _h(t_n)$ is the discretized tumor domain at time $t_n$.

The advection of cell density according to (2.7) is solved by an explicit finite volume (FV) scheme using the pressure distribution at a given time step. We consider a square Cartesian grid and an initial uniform cell density of arbitrary shape placed within an otherwise empty domain where $\rho = 0$. We keep track of the moving boundary by finding volumes where $\rho < \rho _{thresh}$. We apply the pressure boundary condition (2.6) on such volumes that are simultaneously adjacent to volumes with $\rho \ge \rho _{thresh}$ (details on the implementation of surface tension are found in the next paragraph). The threshold was determined to ensure a front speed consistent with the compatibility condition (2.8) during tumor growth and the value $\rho _{thresh} = 0.9$ was chosen to reduce smearing effects close to the boundary and maintain $\rho \approx 1$ across the tumor domain. Furthermore, cell densities outside the tumor boundary should not consume oxygen, and we therefore set $\lambda = 0$ for volumes with $\rho < \rho _{thresh}$. Using a first order upwind scheme to solve (2.7) including a noise term amounts to the following FV scheme

B.2 $$\begin{aligned} \rho _i^{n+1}= &  \rho _i^{n} + \Delta t F_{i}^n + \omega \vert \Delta t F_{i}^n \vert ^{1/2} \times \mathcal {N}, \\ F_{i}^n= &  - \frac{1}{V_i} \sum _{e_{ij} \in \partial V_i} \frac{p_j^n - p_i^n}{h_{ij}}e_{ij} R_{ij}^n + \Gamma _i^{n}, \nonumber \\ R_{ij}^n= &  {\left\{ \begin{array}{ll} \rho _i^n, \quad \text {if } p_i^n - p_j^n \ge 0 \\ \rho _j^n, \quad \text {if } p_i^n - p_j^n < 0 \\ \end{array}\right. }\nonumber \end{aligned}$$

at time $t_n$, where $V_i$ is the volume of the volume element, $\partial V_i$ is the boundary of the volume, $e_{ij}$ is the unique edge adjacent to volumes $V_i$ and $V_j$. The quantities $\rho _i^{n}$ and $\Gamma _i^{n}$ are the volume average quantities of $\rho$ and $\Gamma$, respectively, at time $t_n$ taken over volume $V_i$. Finally, to excite all perturbation modes of the boundary without discretization bias, we have added scaled Gaussian noise to the cell density change as the last term in (B.2) dictates, with $\mathcal {N} \sim \mathcal {N}(0,1)$ and $\omega = 0.025$. The form of the noise is suggested by a Wiener process approximation of a Poissonian interpretation of the drift term in (B.2) at a system size $\omega ^{-1/2}$. For our simulations we used a Cartesian discretization of the mesh with edge length $\Delta x = 0.02$ and $\Delta t = \min (\Delta x, 0.1/\max _i(\vert F_i^n \vert ) )$, which yielded stable solutions across our experiments.

Surface tension The curvature *C* in the pressure boundary condition (2.6) is evaluated numerically on the tumor boundary by estimating the derivatives on the corresponding contour lines. At each time step, we find the contour lines of the tumor boundary where $\rho = \rho _{thresh}$ for the PDE model, and where $u_i = 1$ for the DLCM model. These contour lines are then parameterized by $(x_{c}(s), y_{c}(s))$ for $s \in [0,S]$ assuming periodicity, i.e., $(x_{c}(s), y_{c}(s)) = (x_{c}(s+S), y_{c}(s+S))$, since they are closed curves. We find the cubic spline of each coordinate such that after differentiation we obtain the signed curvature

B.3 $$\begin{aligned} C = \frac{x_{c}(s)'y_{c}(s)'' - x_{c}(s)'' y_{c}(s)'}{(x_{c}(s){'}^2 + y_{c}(s){'}^2)^{3/2}}. \end{aligned}$$

We then calculate the Young-Laplace pressure difference at each boundary volume by using the curvature given by (B.3) at the contour point closest to the volume. We include a simple threshold in our implementation to avoid that a pressure boundary condition is applied to undesired contours. Specifically, we neglect contour lines that consist of a number of points less than a fraction $f_{\min } = 0.95$ of the largest contour. Thus, holes within the tumor or clusters of cells outside the tumor do not produce any surface tension. This prevents spurious surface tension and pressure values, which would otherwise be present in particular in the stochastic model.

DLCM modifications The physics of the PDE model motivates two features of the stochastic mode not present in the original presentation (Engblom et al. 2018), namely surface tension and pressure sinks due to mass loss.

Surface tension is implemented in the same manner in DLCM as for the PDE solver. However, the exclusion of certain events in DLCM due to the voxel carrying capacity introduces a bias associated with the strength of surface tension. Therefore, we allow cells in singly occupied voxels on the boundary to migrate into the population with rate coefficient equal to $D_1$. Without inwards migration, there is nothing to balance out the sporadic outwards migration induced by surface tension on the inherently noisy boundary. In particular, allowing inwards migration ensures that higher levels of surface tension do not introduce a bias in the average front speed of the tumor due to this noise. We note that this bias is unavoidably present in the initial growth phase of the tumor when no attractive, necrotic region exists, as can be observed in the higher initial rate of growth of Fig. 6a compared with Fig. 8a.

The PDE model expresses that mass loss due to cell death produces pressure sinks that drive cell migration to replace the lost mass, and that the pressure sinks are proportional to the rate of mass loss, see (2.10). In the stochastic setting, cell mass loss occurs for necrotic cells that are degrading at the rate $\mu _{\textrm{deg}}$, and for simplicity we use that the pressure sinks and pressure sources are equal to $\mu _{\textrm{death}}$ and $\mu _{\textrm{prol}}$, respectively, akin to the PDE. Further, to ensure that the degrading cells are pushed by the population pressure to the center of the necrotic region before they have fully degraded, we derive an approximate value of the degradation rate $\mu _{\textrm{deg}}$ depending on $\mu _{\textrm{death}}$ for this to happen. Consider a fixed, radially symmetric necrotic region and the motion of a particle from the region’s boundary into its center governed by (2.8). The pressure gradient at any point in this region is found from (3.12) and letting the particle start at $r(t = 0) = r_n$, its position is given by

B.4 $$\begin{aligned} r(t) = r_n e^{-\mu _{\textrm{death}}t/2}. \end{aligned}$$

The expected time $\tau$ it takes for the particle to reach $r(\tau ) = \delta r_n, 0 \le \delta < 1$ of the necrotic region’s center is thus readily obtained from (B.4). The condition on $\mu _{\textrm{death}}$ that ensures that the expected time for the particle to degrade is equal to $\tau$ is then given by $1/\tau$ as

$$\begin{aligned} \mu _{\textrm{deg}}= \frac{\mu _{\textrm{death}}}{2\log (1/\delta )}. \nonumber \end{aligned}$$

For our implementation, we assume that $\delta = 0.01$ is enough to ensure that migration of necrotic cells to the tumor center is possible and occurs frequently.

*PDE effective parameters*

The effective parameters of the PDE model corresponding to the DLCM model parameters are derived by comparing the models radially symmetric solutions. There are three parameters that differ in value due to model differences: $\mu _{\textrm{prol}}$, $\mu _{\textrm{death}}$, and $\lambda$. Firstly, there is a difference in volumetric growth rates between the models since the DLCM tumor must undergo both a proliferation event and a migration event separately to expand in size. From a DLCM model simulation using the parameters in Table 2 and $\sigma = 0$, we consider the initial exponential growth phase ($r_n = r_q = 0$) such that the solution to (3.9) reduces to $r_p(t) = r_p(0) \exp (\mu _{\textrm{prol}}/2 \, t)$. We fit observations of $r_p$ during this phase by minimizing the mean-square error to get an estimate of the corresponding effective growth rate of the PDE model, and we find that $\bar{\mu }_{\textrm{prol}}\approx \mu _{\textrm{prol}}/2.7$. Since the dimensionless parameter $\bar{\mu }_{\textrm{death}}$ is proportional to $\bar{\mu }_{\textrm{prol}}^{-1}$, the former is scaled accordingly. Secondly, we note that since doubly occupied voxels consume twice the amount of oxygen in DLCM, the effective $\lambda$ can be expected to be greater for the corresponding PDE assuming identical characteristic regions. The effective parameter $\bar{\lambda }$ is estimated by taking the mean of (3.3) using the fixed DLCM model parameter $\kappa _{\textrm{prol}}$ and observed values of $(r_n,r_q,r_p)$ during a stationary phase of the DLCM simulation. This gives the estimate $\bar{\lambda } \approx 1.15$. The same procedure yields $\bar{\mu }_{\textrm{prol}}= 1.0$ and $\bar{\lambda } = 1.1$ for the other set of experiments that uses different parameters shown in Fig. 10.

## References

[CR1] Anderson AR et al (2006) Tumor morphology and phenotypic evolution driven by selective pressure from the microenvironment. Cell 127(5):905–915. 10.1016/j.cell.2006.09.042 17129778 10.1016/j.cell.2006.09.042

[CR2] Armbrecht L, Dittrich PS (2017) Recent advances in the analysis of single cells. Anal Chem 89(1):2–21. 10.1021/acs.analchem.6b04255 28105840 10.1021/acs.analchem.6b04255 PMC5642847

[CR3] Drawert B, Engblom S, Hellander A (2012) URDME: a modular framework for stochastic simulation of reaction-transport processes in complex geometries. BMC Syst Biol 6(76):1–17. 10.1186/1752-0509-6-76 22727185 10.1186/1752-0509-6-76 PMC3439286

[CR4] Barbolosi D et al (2016) Computational oncology–mathematical modelling of drug regimens for precision medicine. Nat Rev Clin Oncol 13(4):242–254. 10.1038/nrclinonc.2015.204 26598946 10.1038/nrclinonc.2015.204

[CR5] Bearer EL et al (2009) Multiparameter computational modeling of tumor invasion. Cancer Res 69(10):4493–4501. 10.1158/0008-5472.CAN-08-3834 19366801 10.1158/0008-5472.CAN-08-3834 PMC2835777

[CR6] Brodland GW (2015) How computational models can help unlock biological systems. Semin Cell Dev Biol 47:62–73. 10.1016/j.semcdb.2015.07.001 26165820 10.1016/j.semcdb.2015.07.001

[CR7] Brú A et al (2003) The universal dynamics of tumor growth. Biophys J 85(5):2948–2961. 10.1016/S0006-3495(03)74715-8 14581197 10.1016/S0006-3495(03)74715-8 PMC1303573

[CR8] Byrne HM, Chaplain MA (1996) Modelling the role of cell-cell adhesion in the growth and development of carcinomas. Math Comput Model 24(12):1–17. 10.1016/S0895-7177(96)00174-4

[CR9] Cermak N et al (2016) High-throughput measurement of single-cell growth rates using serial microfluidic mass sensor arrays. Nat Biotechnol 34(10):1052–1059. 10.1038/nbt.3666 27598230 10.1038/nbt.3666 PMC5064867

[CR10] Cristini V, Lowengrub J, Nie Q (2003) Nonlinear simulation of tumor growth. J Math Biol 46:191–224. 10.1007/s00285-002-0174-6 12728333 10.1007/s00285-002-0174-6

[CR11] Cristini V et al (2005) Morphologic instability and cancer invasion. Clin Cancer Res 11(19):6772–6779. 10.1158/1078-0432.CCR-05-0852 16203763 10.1158/1078-0432.CCR-05-0852

[CR12] Davies K et al (2014) On the derivation of approximations to cellular automata models and the assumption of independence. Math Biosci 253:63–71. 10.1016/j.mbs.2014.04.004 24769324 10.1016/j.mbs.2014.04.004

[CR13] Deisboeck TS, Couzin ID (2009) Collective behavior in cancer cell populations. BioEssays 31(2):190–197. 10.1002/bies.200800084 19204991 10.1002/bies.200800084

[CR14] Deisboeck TS et al (2011) Multiscale cancer modeling. Annu Rev Biomed Eng 13:127–155. 10.1146/annurev-bioeng-071910-124729 21529163 10.1146/annurev-bioeng-071910-124729 PMC3883359

[CR15] Drasdo D, Höhme S (2005) A single-cell-based model of tumor growth in vitro: monolayers and spheroids. Phys Biol 2(3):133. 10.1088/1478-3975/2/3/001 16224119 10.1088/1478-3975/2/3/001

[CR16] Engblom S, Wilson DB, Baker RE (2018) Scalable population-level modelling of biological cells incorporating mechanics and kinetics in continuous time. R Soc Open Sci 5(8):180379. 10.1098/rsos.180379 30225024 10.1098/rsos.180379 PMC6124129

[CR17] Fletcher AG et al (2013) Implementing vertex dynamics models of cell populations in biology within a consistent computational framework. Prog Biophys Mol Biol 113(2):299–326. 10.1016/j.pbiomolbio.2013.09.003 24120733 10.1016/j.pbiomolbio.2013.09.003

[CR18] Folkman J, Hochberg M (1973) Self-regulation of growth in three dimensions. J Exp Med 138(4):745–753. 10.1084/jem.138.4.745 4744009 10.1084/jem.138.4.745 PMC2180571

[CR19] Frieboes HB et al (2009) Prediction of drug response in breast cancer using integrative experimental/computational modeling. Cancer Res 69(10):4484–4492. 10.1158/0008-5472.CAN-08-3740 19366802 10.1158/0008-5472.CAN-08-3740 PMC2720602

[CR20] Gerlee P, Anderson AR (2007) Stability analysis of a hybrid cellular automaton model of cell colony growth. Phys Rev E 75(5):051911. 10.1103/PhysRevE.75.051911 10.1103/PhysRevE.75.051911 17677102

[CR21] Giverso C, Verani M, Ciarletta P (2016) Emerging morphologies in round bacterial colonies: comparing volumetric versus chemotactic expansion. Biomech Model Mechanobiol 15(3):643–661. 10.1007/s10237-015-0714-9 26296713 10.1007/s10237-015-0714-9

[CR22] Greenspan H (1976) On the growth and stability of cell cultures and solid tumors. J Theoret Biol 56(1):229–242. 10.1016/S0022-5193(76)80054-9 1263527 10.1016/s0022-5193(76)80054-9

[CR23] Grimes DR et al (2016) The role of oxygen in avascular tumor growth. PLoS ONE 11(4):e0153692. 10.1371/journal.pone.0153692 27088720 10.1371/journal.pone.0153692 PMC4835055

[CR24] Hanahan D, Weinberg RA (2000) The hallmarks of cancer. Cell 100(1):57–70. 10.1016/S0092-8674(00)81683-9 10647931 10.1016/s0092-8674(00)81683-9

[CR25] Hanahan D, Weinberg RA (2011) Hallmarks of cancer: the next generation. Cell 144(5):646–674. 10.1016/j.cell.2011.02.013 21376230 10.1016/j.cell.2011.02.013

[CR26] Jin W et al (2016) Reproducibility of scratch assays is affected by the initial degree of confluence: experiments, modelling and model selection. J Theoret Biol 390:136–145. 10.1016/j.jtbi.2015.10.040 26646767 10.1016/j.jtbi.2015.10.040

[CR27] Lowengrub JS et al (2009) Nonlinear modelling of cancer: bridging the gap between cells and tumours. Nonlinearity 23(1):R1. 10.1088/0951-7715/23/1/r01 10.1088/0951-7715/23/1/r01 PMC2929802 20808719

[CR28] Mather W et al (2010) Streaming instability in growing cell populations. Phys Rev Lett 104(20):208101. 10.1103/PhysRevLett.104.208101 20867071 10.1103/PhysRevLett.104.208101 PMC2947335

[CR29] Oraiopoulou M-E et al (2018) Integrating in vitro experiments with in silico approaches for Glioblastoma invasion: the role of cell-to-cell adhesion heterogeneity. Sci Rep 8(1):1–13. 10.1038/s41598-018-34521-5 30385804 10.1038/s41598-018-34521-5 PMC6212459

[CR30] Quail DF, Joyce JA (2013) Microenvironmental regulation of tumor progression and metastasis. Nat Med 19(11):1423–1437. 10.1038/nm.3394 24202395 10.1038/nm.3394 PMC3954707

[CR31] Roose T, Chapman SJ, Maini PK (2007) Mathematical models of avascular tumor growth. SIAM Rev 49(2):179–208. 10.1137/S0036144504446291

[CR32] Saadatpour A et al (2015) Single-cell analysis in cancer genomics. Trends Genet 31(10):576–586. 10.1016/j.tig.2015.07.003 26450340 10.1016/j.tig.2015.07.003 PMC5282606

[CR33] Saffman PG, Taylor GI (1958) The penetration of a fluid into a porous medium or Hele-Shaw cell containing a more viscous liquid. Proc R Soc A Math Phys Eng Sci 245(1242):312–329. 10.1098/rspa.1958.0085

[CR34] Schlüter DK, Ramis-Conde I, Chaplain MA (2015) Multi-scale modelling of the dynamics of cell colonies: insights into cell-adhesion forces and cancer invasion from in silico simulations. J R Soc Interface 12(103):20141080. 10.1098/rsif.2014.1080 25519994 10.1098/rsif.2014.1080 PMC4305411

[CR35] Scianna M, Preziosi L (2012) A hybrid model describing different morphologies of tumor invasion fronts. Math Model Nat Phenom 7(1):78–104. 10.1051/mmnp/20127105

[CR36] Szabó A, Merks RM (2013) Cellular potts modeling of tumor growth, tumor invasion, and tumor evolution. Front Oncol 3:87. 10.3389/fonc.2013.00087 23596570 10.3389/fonc.2013.00087 PMC3627127

[CR37] Valero C et al (2018) Combined experimental and computational characterization of crosslinked collagen-based hydrogels. PLoS ONE 13(4):e0195820. 10.1371/journal.pone.0195820 29664953 10.1371/journal.pone.0195820 PMC5903660

[CR38] Walker BJ et al (2023) Minimal morphoelastic models of solid tumour spheroids: a tutorial. Bull Math Biol. 10.1007/s11538-023-01141-8 10.1007/s11538-023-01141-8 PMC10060352 36991173

[CR39] Whitaker S (1986) Flow in porous media I: A theoretical derivation of Darcy’s law. Transp Porous Media 1(1):3–25. 10.1007/BF01036523

[CR40] Yin A et al (2019) A review of mathematical models for tumor dynamics and treatment resistance evolution of solid tumors. CPT Pharmacomet Syst Pharmacol 8(10):720–737. 10.1002/psp4.12450 10.1002/psp4.12450 PMC6813171 31250989

